# Fluorescent optotracers for bacterial and biofilm detection and diagnostics

**DOI:** 10.1080/14686996.2023.2246867

**Published:** 2023-09-05

**Authors:** Agneta Richter-Dahlfors, Elina Kärkkäinen, Ferdinand X. Choong

**Affiliations:** a AIMES – Center for the Advancement of Integrated Medical and Engineering Sciences at Karolinska Institutet and KTH Royal Institute of Technology, Stockholm, Sweden; b Department of Neuroscience, Karolinska Institutet, Stockholm, Sweden; c Department of Fiber and Polymer Technology, School of Chemistry, Biotechnology and Health, KTH Royal Institute of Technology, Stockholm, Sweden

**Keywords:** Microbes, fungi, amyloid, polysaccharides, cellulose, antimicrobial susceptibility testing, AST, optotracing, EbbaBiolight, Carbotrace

## Abstract

Effective treatment of bacterial infections requires methods that accurately and quickly identify which antibiotic should be prescribed. This review describes recent research on the development of optotracing methodologies for bacterial and biofilm detection and diagnostics. Optotracers are small, chemically well-defined, anionic fluorescent tracer molecules that detect peptide- and carbohydrate-based biopolymers. This class of organic molecules (luminescent conjugated oligothiophenes) show unique electronic, electrochemical and optical properties originating from the conjugated structure of the compounds. The photophysical properties are further improved as donor-acceptor-donor (D-A-D)-type motifs are incorporated in the conjugated backbone. Optotracers bind their biopolymeric target molecules via electrostatic interactions. Binding alters the optical properties of these tracer molecules, shown as altered absorption and emission spectra, as well as ON-like switch of fluorescence. As the optotracer provides a defined spectral signature for each binding partner, a fingerprint is generated that can be used for identification of the target biopolymer. Alongside their use for *in situ* experimentation, optotracers have demonstrated excellent use in studies of a number of clinically relevant microbial pathogens. These methods will find widespread use across a variety of communities engaged in reducing the effect of antibiotic resistance. This includes basic researchers studying molecular resistance mechanisms, academia and pharma developing new antimicrobials targeting biofilm infections and tests to diagnose biofilm infections, as well as those developing antibiotic susceptibility tests for biofilm infections (biofilm-AST). By iterating between the microbial world and that of plants, development of the optotracing technology has become a prime example of successful cross-feeding across the boundaries of disciplines. As optotracers offers a capacity to redefine the way we work with polysaccharides in the microbial world as well as with plant biomass, the technology is providing novel outputs desperately needed for global impact of the threat of antimicrobial resistance as well as our strive for a circular bioeconomy.

## IMPACT STATEMENT

Optotracing is the first, and so far, the only, available technique for monitoring dynamic biofilm formation and differentiation between the EPS constituents under live conditions. This opens for development of intravital probes that will allow us to study biofilm development in vivo and the role of biofilm on infection outcome. Since the first publication of the use of optotracers to study the formation of biofilms by Salmonella, the field has expanded to include the study of a wide range of pathogenic microbes as well as its use in diagnosing biofilm infectiuons. This review collects important findings of research that apply optotracing, in order to provide a single article that documents the applications of this exciting technology.

## GRAPHICAL ABSTRACT

**Figure:**
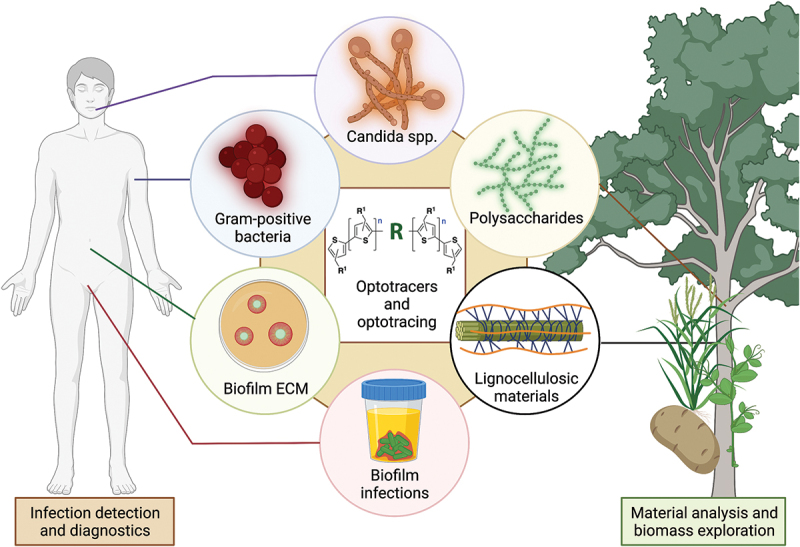

## 1. Introduction

Infectious diseases pose a massive risk to public health and the global economy, as highlighted by the recent SARS-CoV-2 coronavirus pandemic. For bacterial infections, antibiotics remain the mainstay treatment strategy. Numerous classes of compounds exist, bringing about bactericidal or bacteriostatic effects by disrupting the integrity of a bacterium’s physiology or normal functioning of metabolic pathways. Unfortunately, a history of sub-optimal, off-label and excessive use of antibiotics has led to the evolution of resistance mechanisms in bacteria. The development of antimicrobial resistance (AMR) has severe consequences to the infected individual, resulting in inefficient extermination of diseases leading and/or contributing to prolonged illness, disability, and death [1–5]. A classic example is the broad-spectrum antibiotics, such as amoxicillin and ciprofloxacin, that are used in frontline treatment until the causative pathogen has been identified and the most effective narrow-spectrum antibiotic has been determined and prescribed to the patient [6]. While broad-spectrum antibiotics are effective against a wide range of pathogens, research has shown them to be strong drivers of AMR as well [6]. Also, some antibiotics, such as fluoroquinolones, can induce resistances to other antibiotic classes [7]. Yet an exacerbating, but often overlooked, factor is that circa 80% of all chronic and recurrent microbial infections are associated with the special microbial lifestyle termed biofilm [8]. As the biofilm provides protection and evasion from environmental insults, including antimicrobial drugs, the presence of this unique lifestyle needs to be determined in order to effectively treat the patient and reduce the global AMR burden [9,10].

The severity of AMR was illustrated in a recent review in the Lancet, showing that 4.95 million deaths in 2019 were associated with AMR bacteria, making it a leading cause of death worldwide [11]. The World Bank Group estimates that a continued rise in antimicrobial resistance by year 2050 will lead to 10 million deaths annually, and an associated reduction of 2%−3.5% in Gross Domestic Product (GDP) [11–13]. Beyond its direct impact, AMR will also undermine the progress in other medical fields, such as oncology and orthopedics, with any bacterial contamination becoming life-threatening. Unfortunately, the attempts to develop new antibiotics and the prevention and management of AMR has not yet shown any effects on the reduction of multidrug-resistant isolates worldwide.

Mitigation of infections is critically dependent on early detection and correct diagnosis of the pathogen. Unfortunately, current detection and diagnostic methods are often restricted to days-long waits for bacteria to grow [14,15]. This delay is particularly detrimental for patients symptomatic of sepsis, wherein knowledge about causative microbial pathogens is essential to rapidly prescribe adequate antimicrobial therapy [16–18]. Simple, rapid, and sensitive analytical methods for point-of-care (POC) diagnosis have been increasingly demanded in recent decades [19]. Such devices are especially relevant for rapid response and management during outbreaks of zoonotic pathogens which traverse the species barrier and have high epidemic potential [20–24]. Few occasions in modern history have been more pressing for developments in this field than the present SARS-CoV-2 coronavirus pandemic, which in the period of December 2019 to May 2021 infected more than 160 million people in over 210 countries and territories, resulting in more than 3.3 million deaths [25]. Within the landscape of this pandemic, it has become clear that rapid POC diagnostic testing combined with smart and data-driven management of behavioral guidelines is paramount to reduce the spread of infection and ease the impact on health systems. Unfortunately, rapid molecular testing is still relatively expensive and methodologically difficult for less developed healthcare systems [26,27].

At present, clinical diagnostics of bacterial infections relies on culturing methods that promote growth of the microbe and/or isolation of the causative pathogen from the sample. The Gram stain is often used initially to gather preliminary information about the pathogen based on the color and morphology of bacterial colonies [28]. To obtain specific details pertaining to the pathogen’s species and virulence information, more costly molecular biology techniques are used, such as polymerase chain reaction (PCR), quantitative PCR (qPCR), fluorescence *in situ* hybridization (FISH), and immunoassays such as enzyme-linked immunosorbent assay (ELISA) [29–31]. With the exception of PCR, these methods tend to be time-consuming and only applicable for the viable and culturable subset of bacteria [32]. Also, these analysis only generate part of the information necessary for prescription of appropriate treatment, supplementary procedures, such as antimicrobial susceptibility testing (AST) must be used to identify the antibiotic that would be most effective in clearing the infection [33–35]. Optimal treatment regimens, requiring timely and accurate identification of the causative pathogen and its antibiotic susceptibility pattern, are crucial to hinder further AMR development [36–38].

AST is instrumental in the development of new effective antimicrobial compounds. Conventional ASTs, exemplified by the prevailing microbroth dilution assay and the Kirby-Bauer disc diffusion test, are based on monitoring bacterial growth and identifying inhibition zones with and without antibiotics [39–41]. These methods are relatively simple to perform, easy to interpret, accurate and cost-effective [33,42]. However, a downside is that these methods are time-consuming, labor-intensive, and require skilled manpower [41]. Recently, much attention has been on developing rapid AST techniques with increased throughput and capacity for automation, to allow POC identification of antibiotic susceptibilities and AMR. Novel technologies and big-data approaches, such as matrix-assisted laser desorption, ionization time-of-flight mass spectrometry (MALDI-TOF MS) and next-generation sequencing (NGS) are being introduced into the toolbox for microbial diagnostics [43,44]. MALDI-TOF MS provides fast analysis of microbial samples taking only a few minutes, as well as the prospect of determining AMR profile of pathogens on which active research is being conducted [45,46]. However, the method holds the large drawback of the need for pre-culturing of pure cultures, thereby reducing the convenience and speed of the technique [45,47]. NGS provides the great advantage of identifying non-culturable bacterial species and is increasingly being applied in microbial diagnostic laboratories for identification of pathogens based on 16S rRNA sequencing [48,49]. Furthermore, characteristics such as virulence- and AMR genes can be identified using metagenomic approaches [49]. While NGS does not require culturing, time-consuming pre-processing, such as library preparation is needed, and the extensive data analysis is demanding [50,51]. Whereas MALDI-TOF MS and NGS are compelling and powerful techniques, they both require skilled personnel and special, most often expensive equipment. As such, neither one of these techniques are commonplace.

Here, we describe recent research on optotracing, a direct sensing technology that opens new and exciting opportunities for infection research, diagnostics, and treatment. As a non-toxic, culture-independent method, optotracing is demonstrated to rapidly discriminate gram-positive from gram-negative bacterial species, and to provide unprecedented information on the bacterial biofilm lifestyle. The latter is translated to clinical situations, as optotracing is developed into the first method able to test the effects of antibiotics on bacteria in the biofilm lifestyle, as well as to diagnose a biofilm infection. A main feature of optotracing is to detect and discriminate polysaccharides, irrespective of source. The review therefore devotes the final section to describe the interdisciplinary development of optotracing, iterating between infection research and plant science. Serving as an enabling technology, equally fit to target problems related to bacterial infections as well as to aid in our strive towards production of fossil free materials, the optotracing technology may help to find solutions not only to combat the challenges posed by infections, but also to promote a shift towards a sustainable, circular bioeconomy.

## 2. Bacterial sensing techniques

Among the new technologies devised to enable rapid detection of pathogenic bacteria are various forms of biosensors [52]. Biosensors are traditionally defined as analytical appliances whose functionality is based on a catalytic or affinity-based biochemical interaction of a biorecognition element, such as natural- or engineered receptors, with target component(s). This interaction generates a signal that is converted into measurable readouts by a transducing element [53–56]. As traditional biosensors are relatively difficult to assemble and biorecognition elements tend to be temperature- and photo-sensitive, a need for new, direct sensing technologies has been expressed, as the simplicity of such methods will open for new possibilities [57]. These new methods for direct biosensing could revolutionize the field as they simplify the multicomponent system of biosensing, thereby presenting huge potential for new versatile applications.

As direct sensing combines the recognition component and transducer in one and the same element, such sensors are often built using conducting polymers. This material holds several superior features, such as electronic- and ionic conductivity, a polymeric structure, and exposure of a large surface area. An electrochemical sensor based on the conductive polymer PEDOT:PSS has been developed in order to enable direct detection of bacterially secreted redox-active compounds [58]. Further development of a transistor-based design with a PEDOT:PSS channel generated a possibility to achieve bacterial sensing during growth in real-time, as redox active species were produced by bacteria [59].

## 3. Design and synthesis of thiophene-based optotracers for direct sensing of bacteria

Thiophene-based ligands have been developed as a class of fluorescent tracer molecules for optical assignments of various biopolymers, ranging from disease-associated protein aggregates, bacterial amyloids as well as polysaccharides [60]. Here, we provide a general background to the optical properties of conjugated polyelectrolytes (CPEs), followed by an overview of the chemical evolution of luminescent conjugated polythiophenes (LCPs), via luminescent conjugated oligothiophenes (LCOs) and their donor-acceptor-donor (D-A-D) counterparts, to optotracers used for bacterial detection. Readers with special interest in this development are referred to an extensive review on this topic, recently published by the group of K. P. R. Nilsson, a leading expert in the field [61].

**Figure 1.**
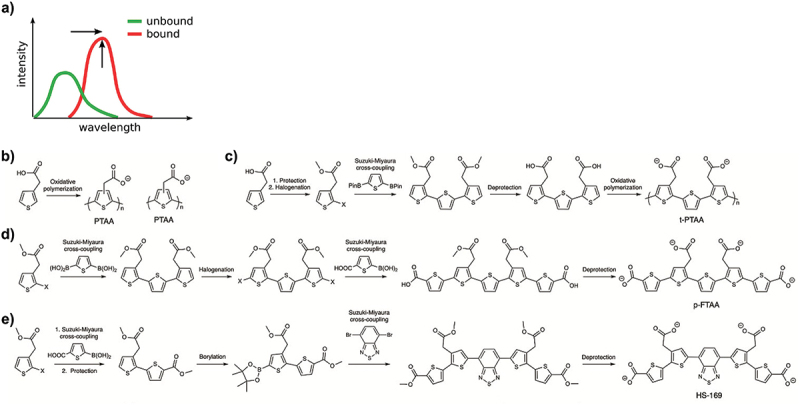
(a) Schematic representation of changes in fluorescence intensity and red-shift of the spectrum depending on the backbone conformation. Reprinted from Löffler et al. 2019 and Butina et al. 2022 [60,63], (open access CC by 4.0). (b-e) general synthetic routes of luminescent conjugated polythiophenes (LCPs) represented by (b) PTAA, and (c) t-PTAA. (d) general synthetic route for achieving chemically defined luminescent conjugated oligothiophenes (LCOs) represented by the anionic pentameric LCO p-FTAA. (e) general synthetic route for achieving chemically defined donor-acceptor-donor (D–A –D) based LCOs represented by HS-169. Panels reprinted from Björk et al. 2023 [61], (open access CC BY-NC 4.0).

The optical detection scheme of thiophene-based ligands is based on the intrinsic conformation sensitive optical properties unique to this class of molecules. Key to this is the π-conjugated backbone. This enables excitation of electrons from the highest occupied molecule orbital (HOMO) in the π-band to the lowest unoccupied molecule orbital (LUMO) in the π* band. Energy in the range of the UV–vis spectrum is required for π-π* transitions. Following excitation, electrons relax by emission, whereby photons give rise to fluorescence or to energy transfer to acceptors, which are events that can be interpreted by UV–vis spectroscopy. Yet an important molecular feature is the ionic pendant groups. These side chains confer water solubility, and importantly, allow the formation of electrostatic interactions with oppositely charged macromolecules [62]. Upon binding, the flexible conjugated thiophene backbone distorts in response to the non-covalent electrostatic interactions. Binding interactions lead to the flattening of the molecular backbone and a more effective conjugation, which is readily observed as a red-shift in the fluorescence excitation and/or increase in fluorescence emission intensity (Figure 1(a)). This conformation-dependent optical detection principle provides target-specific spectral signatures, which are used for optical detection of biomacromolecules.

Originally, LCPs were used for biosensing purposes. These molecules consist of several thiophene units with different side-chain functionalities along the conjugated thiophene backbone (Figure 1(b)). Taking advantage of their excellent photophysical properties, LCPs were used as optical biosensors for biological processes, such as DNA hybridization and ligand–receptor interaction [64–68]. It was next found that the spectral signature from zwitterionic and anionic LCPs could be used for the detection of conformational changes in synthetic peptides, for detection of amyloid fibril formation *in vitro*, as well as staining and differentiation of protein aggregates in tissue sections [69–75]. However, as oxidative polymerization of thiophene monomers render a polydisperse material with different thiophene-chain lengths and no regioregularity of the side-chain functionalities (Figure 1(b)), a second generation, slightly more chemically defined LCPs was generated by oxidative polymerization of trimer-building blocks, which were created by a palladium mediated Suzuki-Miyaura cross-coupling reaction of a mono-halogenated thiophene unit with a di-borylated thiophene (Figure 1(c)) [76,77]. While this scheme generated regiospecific ligands such as t-PTAA (Figure 1(c)), the chain length still varied between 9 and 12 thiophene units. To overcome this variability, K.P.R. Nilsson and co-workers designed and synthesized chemically defined anionic pentameric oligothiophenes based on a halogenation of the trimer building block followed by palladium mediated Suzuki-Miyaura cross-coupling reaction of the trimer to borylated thiophene monomers (Figure 1(d)) [78]. Several other chemically defined tetrameric, pentameric, hexameric, and heptameric LCOs have been generated by similar synthetic routes, using palladium mediated Suzuki-Miyaura cross-coupling reactions of different halogenated or borylated thiophene building blocks [78–85]. To further extend the photo-physical properties vital to multiplex detection methodologies, pentameric, and heptameric D–A–D based LCOs were synthesized by replacing the central thiophene unit with other heterocyclic moieties, such as benzothiadiazole (BTD) or quinoxaline [86,87]. In D–A–D based LCOs, the di- or tri-thiophene building blocks act as donors, whereas the BTD or quinoxaline moiety is the acceptor. The D–A–D based ligands were assembled in palladium-mediated Suzuki – Miyaura cross-coupling reactions of different halogenated or borylated heterocyclic building blocks (Figure 1(e)). The excitation and emission characteristics of D–A–D ligands differ vastly from LCOs lacking these motifs. Accordingly, the photophysical properties of thiophene-based ligands are further broadened, thereby enabling their use in a variety of imaging techniques with different modes of detection.

The chemical evolution of polydispersed to chemically defined thiophene-based ligands has been driven by a need to expand the toolbox of fluorescent ligands for optical assignment of disease-associated protein aggregates. As the technology expanded from this field into others, and became commercially available, the nomenclature changed from the chemical designation of the molecules towards their function. Thus, LCOs are from here onwards designated optotracers, and their application as optotracing, as their uses in bacterial and biofilm detection and diagnostics are reviewed.

The fluorescence-based optotracing technology represents a new direct sensing technology in which detection and reporting components are inherently present within the same molecule. Optotracers typically interact with bacterial cells and/or bacterially produced biopolymers, such as amyloid fibrils and polysaccharides [88–91]. Via its conjugated backbone and/or pendant sidechains, optotracers bind their target biomacromolecules via non-covalent hydrophobic and electrostatic interactions [63,81]. Binding is associated with changes in the flexible conjugated thiophene backbone. As this alters the optotracers’ optoelectronic properties, target-specific spectral signatures revealed by a red-shift in the fluorescence excitation and increase in fluorescence emission intensity, are obtained of each binding event [60,81,88,92].

The new generation of optotracers containing D-A-D motifs present an ON-like switch (on-switch) of fluorescence when the molecule binds to its target [86]. The optotracer emits light of very low intensities in unbound state, but as a target polymer appears to which the optotracer binds, the signal is switched on. The appearance of a target thus acts as a light switch. Together with the notion that optotracers do not show any toxicity towards eukaryotic and prokaryotic cells, the light switch feature provides a unique opportunity to monitor kinetic processes in biological systems in real-time. Monitoring of the bacterial production of polysaccharides and amyloid proteins, both notorious for lacking unique epitopes necessary for generating antibodies for detection can easily be achieved by optotracing, and the technology has thus become commercially available [93,94]. The optotracing technique shows excellent potential as a versatile tool for the detection and diagnosis of bacteria and bacterial biofilms by virtue of the optotracers’ unique configuration [60,63].

## 4. Optotracing in infection research and diagnostics

### 4.1. Detection and growth determination of gram-positive bacteria

Gram-positive bacteria are of significant concern in healthcare. Most gram-positive infections are caused by commensal bacteria that are found naturally on epithelial tissues, such as the skin, mucus membranes, and the gastrointestinal tract [95]. *Staphylococcus aureus* (*S. aureus*) has gained notoriety as a problematic commensal pathogen, particularly when strains gain resistance to multiple antibiotics. Referred to as Methicillin-resistant *S. aureus* (MRSA) infections, these infections impose an estimated annual burden of $28.4 billion in hospitals and $12.4 billion in society, alongside early death and lost productivity [96].

**Figure 2.**
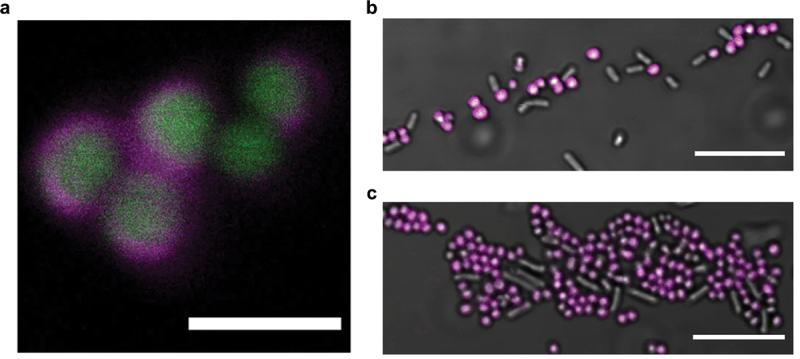
(a) confocal fluorescence micrograph showing optotracer staining of GFP-expressing *S. aureus* 8325–4. The GFP (green) is located in the bacterial cytoplasm, while the optotracer is clearly located to the cell wall (magenta). Scale bar = 2 µm. (b,c) confocal fluorescence micrographs showing selective reporting of gram-positive bacteria (coccoid) in mixes also containing a gram-negative (rod-shaped) species. Selective staining of (b) *S. aureus* when mixed with *Salmonella* Enteritidis and (c) *Staphylococcus epidermidis* when mixed with *E. coli*. Scale bar = 10 µm. Reprinted from Butina et al. 2020 [91], (open access CC by 4.0).

Answering to the need for rapid and more accurate detection and diagnostic tools for gram-positive bacterial infections, optotracing has been shown useful for this category of bacteria [91]. In contrast to gram-negative bacterial species, which produce numerous extracellular biopolymers serving as potential targets for optotracer binding, it is less clear what extracellular polymeric materials gram-positive *S. aureus* expresses [97]. The well-defined and conserved cell wall of gram-positive bacteria was investigated as a potential target for optotracers [91]. The major constituent of gram-positive bacterial cell walls is peptidoglycan, which consists of repeating β(1–4) linked linear units of an N-acetylglucosamine (NAG) – N-acetylmuramic acid (NAM) disaccharide [97,98]. These disaccharide units are in turn cross-linked via pentapeptide chains [99]. Application of a panel of optotracers to a clinical *S. aureus* strain resulted in the characteristic on-switch of the optotracers’ fluorescence and broad patterns of absorption and emission that were distinct to the unbound state [91]. *S. aureus* cells stained by optotracers were also visible under confocal laser scanning microscopy (CLSM) and super-resolution imaging, with the signal clearly localized to the surface of cells (Figure 2(a)) [91]. These changes in optotracer fluorescence did not manifest in planktonic samples of gram-negative bacteria, exemplified by *Escherichia coli* (*E. coli*). This was because gram-negative species lack the thick cell wall, instead they express a dense layer of lipopolysaccharides (LPS) to which optotracers do not bind. This explains why the optotracers were able to selectively illuminate gram-positive bacteria in heterogeneous mixtures of gram-positive and gram-negative bacteria (Figure 2(b)) [91]. Collectively, these results indicated that the optotracers were effective in detecting gram-positive bacteria.

Extending the analysis to *Staphylococcus epidermidis* (*S. epidermidis*), a commensal representative of gram-positive bacteria, revealed identical optical signal qualities to that of *S. aureus* (Figure 2(c)). Further comparisons of recorded signals between the two strains revealed weaker correlation between optotracer fluorescence and culture density, suggesting a weaker or less productive binding of the optotracer to *S. epidermidis* than to *S. aureus*. In contrast, optotracer exposure of cultures of *Enterococcus faecalis* (*E. faecalis*), another gram-positive commensal bacterial species, did not show distinct fluorescence and accordingly, cells remained invisible in CLSM [91]. When analyzing the physical differences between the bacterial strains, the BATH assay (Bacterial Adhesion To Hydrocarbons) showed *S. aureus* to be the strain with most hydrophobic cell envelope, *S. enteritidis* was the most hydrophilic, and *E. faecalis* was an intermediate between the two. With that in mind, altering the surface hydrophobicity of bacteria by changing salt concentrations and pH of the assay buffer was applied to analyze its effect on optotracer binding to bacteria [91]. Under acidic conditions, positive detection of *E. faecalis* by both spectroscopy and CLSM was now possible. These findings indicated that the optotracing methodology could be used to differentiate between gram-positive bacterial strains, and that surface hydrophobicity was a strong determinant to that effect.

**Figure 3.**
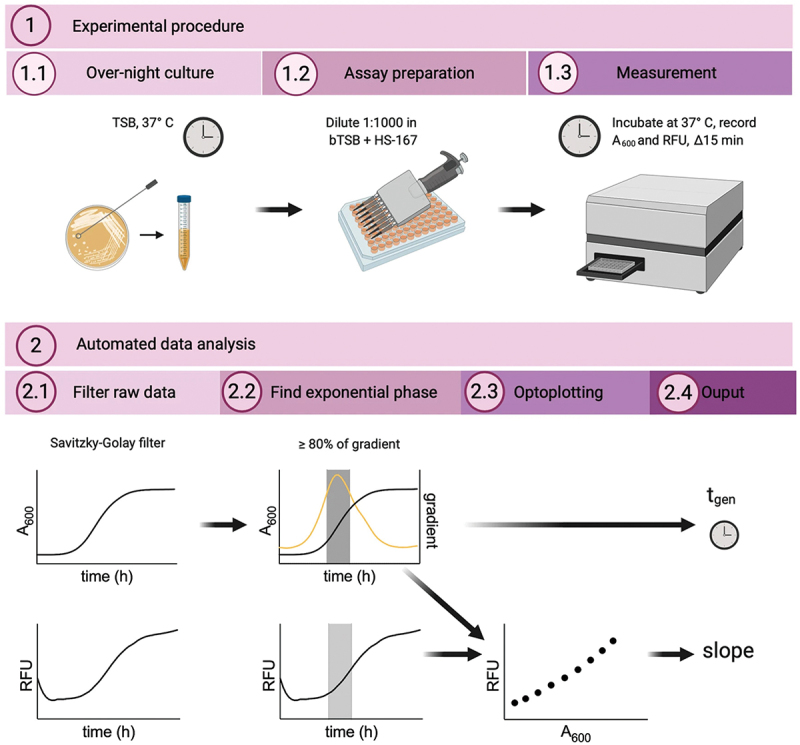
The workflow of automated, high-throughput optotracing analysis based on real-time recordings in live bacterial cultures. The workflow consists of: 1. Experimental procedure and 2. Automated data analysis. The figure was created with Biorender.com. Reprinted from Butina et al. 2020 [91], (open access CC by 4.0).

The oligothiophene backbone of optotracers can be varied such that the central thiophene unit is replaced by heterocyclic motifs. By analyzing the signal quality of such optotracers interacting with *S. aureus*, it was shown that the presence of a central quinoxaline motif resulted in the most distinct signal of bound optotracers compared to the unbound state [91]. These findings were also a strong indication of the benefits in spectral separation and sensitivity brought about by heterocyclic motifs conferring a donor-acceptor-donor (D-A-D) type electronic structure in the optotracers’ conjugated backbone [63,86,100]. In order to identify the binding target of the optotracer on *S. aureus* cells, a genetic approach was taken, in which an unbiased screen of approximately 2000 isogenic strains of a *S. aureus* transposon library were used, in which each strain harbored a well-defined mutation in a nonessential gene [101]. Finding that the optotracer bound all strains within the transposon library implied that the binding target was likely the product of essential gene(s) [91]. Comparative analysis of fluorescence signals between strains revealed, however, instances of attenuated fluorescence, of which >60% were related to genes involved in the formation of the cell envelope. Optotracing of purified preparations of cell envelope components showed positive binding to peptidoglycan and lipoteichoic acid, whereas LPS from gram-negative bacteria remained unstained as expected. This was strong evidence pointing to the cell wall as a target for optotracers. Necessitated by the large pool of samples and the considerable amount of data within absorption and emission patterns, an efficient workflow using algorithm-based analytics had to be developed to enable automated data analysis (Figure 3). In this workflow, a bacterial culture was prepared by re-inoculating an overnight culture into fresh growth media supplemented with the optotracer. As the culture was incubated, culture density was monitored by traditional absorbance recordings as well as recording of the intensity of the fluorescence signals from the optotracer bound to *S. aureus* cells. Extracted data were then processed by a custom-made script which calculated the generation time and correlated fluorescence intensity with the measured absorbance. Given that the correlation was linear, the value of the slope of the correlation curve could be used to guide the evaluation of optotracer binding throughout different growth phases without influencing the growth conditions [63]. This generated a possibility to perform continuous monitoring, which represents an important step towards the translation of optotracing methodologies into bedside diagnostic tools, since the momentous state of the sample can be evaluated conveniently and without the necessity of an external reference.

Key characteristics in the optotracers’ chemical structure of importance for selective detection of gram-positive bacteria have been profiled and related to its photophysical properties. When studying a large panel of optotracers against *S. aureus* (gram-positive) and *Salmonella* Enteritidis (gram-negative) strains, the photophysical properties of each bound and unbound optotracer were evaluated by analyzing the fluorescence intensities of the spectra in so-called spec-plots, as well as the shifts of the wavelengths of the excitation and emission peaks [63]. Comparing tetra-, penta-, hexa-, and heptameric optotracers, the study showed that incremental increases in the length of the thiophene backbone positively correlated with detection of gram-positive bacteria, in which the largest spectral shift and fluorescence intensity was produced by the heptameric optotracer [63]. Since non-covalent binding interaction between aromatic motifs and sugars is well document and understood, it was conceivable that an increased number of thiophene rings would strengthen the binding of optotracers to cell wall polysaccharides through hydrophobic interactions [63,102]. Since most gram-positive bacteria possess an overall charge at neutral pH due to the omnipresent cell wall components peptidoglycan, teichoic acids and other surface presented macromolecules, optotracer binding would naturally be affected by the total charge of the tracer molecule [103]. Experiments showed that heptameric molecules with six negative charges performed poorly in selectively detecting gram-positive, but not gram-negative, bacteria compared to optotracers with four negative charges [63]. Consistently, pentameric molecules with the lowest negative charge (−2) performed better than those with more negative charges (−4) [63]. Taken together, the analysis implied that any addition of charged groups to the molecular structure of the optotracer would distinctly affect the binding and selective detection of bacterial species. Extending this observation, it was also probed for any relationship between the distribution of charges on the optotracers. Despite having a significant effect on optotracer geometry, the arrangement of charged groups along the conjugated backbone did not show any effect on *S. aureus* detection [63]. Collectively, these findings demonstrated the possibility to use informed and directed chemical modification of optotracers to obtain selective detection of gram-positive bacteria, thereby laying the foundation for optotracing as a technique for rapid identification of different bacteria species.

### 4.2. Detection of the biofilm lifestyle of gram-negative bacteria

Biofilms are a natural microbial lifestyle representing highly organised microbial communities. In biofilms, cells are organized into a macrostructure composed of extracellular matrix (ECM) substances, which protect microorganisms from harsh physical, chemical, and nutritional conditions [104–106]. Biofilms are known to form in liquid environments, as well as at air–liquid, air-solid, and liquid–solid interfaces [107]. It is estimated that the cellular content of biofilms can be as low as 10% of the total mass. The extracellular material that encases the bacterial cells can be self-produced by the microbe and/or acquired from its immediate microenvironment [108]. The ECM can be a conglomeration of biopolymers belonging to all four classes of biological macromolecules: carbohydrates, lipids, proteins, and nucleic acids [109]. The complex structures generate specialized microenvironments in the ECM, allowing the creation of synergistic microconsortia of cells with capabilities exceeding those of planktonic cells [110]. In biofilms of enteric bacteria such as Salmonella *spp*. and *E. coli*, key ECM constituents are the amyloid protein curli and the polysaccharide phosphoethanolamine cellulose [111,112].

**Figure 4.**
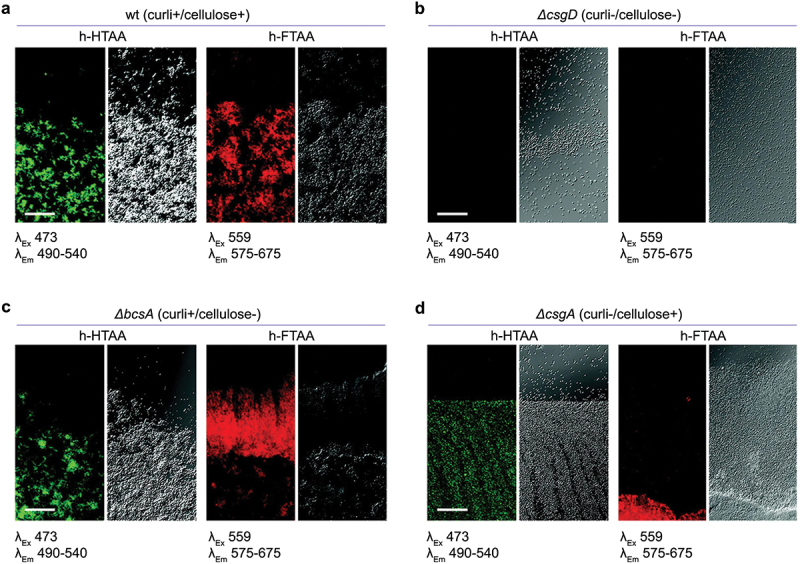
Optotracer staining patterns distinguish different types of *Salmonella* biofilms. (a – d) images from fluorescence confocal microscopy (left) collected at indicated excitation and emission wavelengths, and from transmission confocal microscopy (right) of h-HTAA- and h-FTAA-stained biofilms from a set of isogenic strains of *Salmonella* 3934 with ECM-curli and cellulose phenotypes as indicated. (a) wild type, (b) *ΔcsgD*, (c) *ΔbcsA* and (d) *ΔcsgA* Single optical sections are shown. Scale bar = 50 μm. Reprinted from Choong et al. 2016 [90], (open access CC by 4.0).

Transdisciplinary use of optotracers in microbiological applications was pioneered by Choong *et al.* who applied these tracer molecules to study biofilms formed by the food-borne pathogen *Salmonella* Enteritidis (*S*. Enteritidis) [90]. Optotracer staining of biofilms formed by this strain at the air-liquid interface of a glass slide revealed distinct fluorescence signals (Figure 4(a)). Taking a genetic approach based on a selection of isogenic mutants with deficiencies in the production of cellulose (Δ*bcsA*), curli (Δ*csgA*) and both these ECM components (Δ*csgD*), it was shown that the optotracer signal originates from interaction with amyloid curli fibrils and cellulose in the ECM (Figure 4(b–d)).

**Figure 5.**
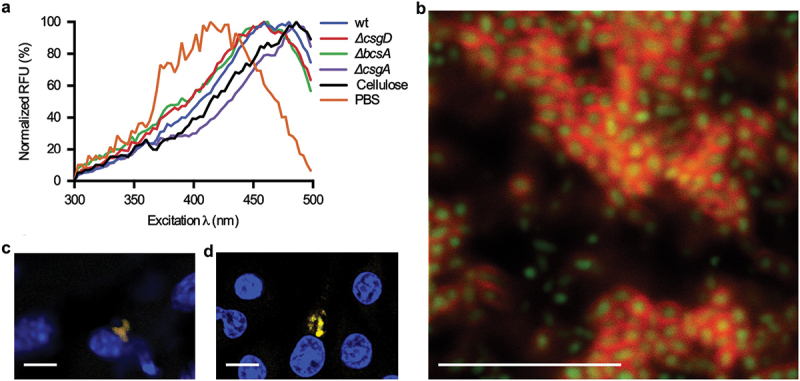
Optotracing differentiates biofilms according to ECM components and detects *in vitro* as well as intracellular biofilms. (a) normalized spectra of h-FTAA mixed with re-suspended biofilm colonies harvested from *Salmonella* 3934 wild type (wt), *ΔcsgD*, *ΔbcsA* and *ΔcsgA* grown for 48 h on LB agar without salt, with emission read at 545 nm. h-FTAA mixed with cellulose and PBS were assayed in parallel for reference. (b) fluorescence confocal microscopy of an unfixed, live biofilm formed by the GFP-expressing *Salmonella* strain 3934 wt stained with h-FTAA. GFP-expressing bacteria (green) are surrounded by the extracellular matrix to which the optotracer binds (red). (c, d) fluorescence confocal microscopy of *S. typhimurium* 14028 *ssaG*:gfp+ (green) infecting (c) the macrophage cell RAW264.7 and (d) the liver in mouse. The optotracer stain shows that cellulose is expressed (red), Hoechst 33,324 shows cell nuclei (blue). Single optical sections are shown. Scale bar = 10 μm. Reprinted from Choong et al. 2016 [90], (open access CC by 4.0).

When analyzing the photophysical properties of bound optotracers by fluorescence spectroscopy, unique absorption and emission patterns were observed for the bound optotracer, which were distinctly different from those of the optotracer in its unbound state [90]. One optotracer exhibited multiple absorption patterns, each representing the expression pattern of ECM components specific for the wild type (wt) isogenic mutants (Figure 5(a)). To achieve spatial details of the location of optotracer binding in the biofilm, confocal laser scanning microscopy was applied to a *Salmonella* strain expressing the green fluorescent protein (GFP). Confocal microscopy revealed large communities of distinct rod-shaped bacteria, surrounded by dense mesh-like structures (Figure 5(b)) [90]. This qualified the selectivity of the tracer molecules to the biofilm ECM. Similar findings in biofilms formed by *Salmonella typhimurim* (*S. typhimurium*) and uropathogenic *E. coli* (UPEC) suggested that ECM-based identification of biofilms was species independent, demonstrating the suitability of optotracing for the study of biofilm formed by a variety of microbes [90,113,114].

Optotracing has been shown to serve to advance our knowledge of the role of biofilms in host–pathogen interactions [90]. *S. typhimurium* is a facultative intracellular pathogen that proliferates in a variety of host cells. Application of an optotracer to infected epithelial cells and macrophages could for the first time demonstrate that bacteria forms biofilm as part of their intracellular lifestyle (Figure 5(c,d)). Identification of intracellular cellulose expression by optotracing strongly implicates an important role for this unique bacterial lifestyle in infection and pathogenicity [115].

By adding optotracers to the culture medium, the optotracer functions as a continuous tracer of ECM production and composition in real-time. Spectrophotometric recordings of a growing *Salmonella* culture revealed a gradual non-linear increase in signal intensity as the culture grew over time [90]. Correlating the optotracer’s signals to culture density showed that ECM production tightly correlates to specific metabolic states, as it initiates at the late exponential/early stationary phase [90,116,117]. Biofilms at the air-solid interface showed the same pattern, suggesting that metabolic control of ECM formation in biofilms is independent of the state of the growth environment [114]. By providing direct, kinetic, phenotypic evidence, complementing genetic evidence of how biofilms form, these studies demonstrated an unprecedented use of optotracers to enable direct real-time monitoring of biofilm formation, which was clearly distinguishable from planktonic growth [90,114]. As optotracers are non-toxic to bacterial systems, these studies revealed the great potential for optotracing as an invaluable tool to detect and monitor biofilms in live systems.

### 4.3. Detection of fungal biofilms

Compounding the global disease burden caused by bacterial infections, fungal pathogens are responsible for close to 13 million infections and 1.5 million deaths annually [118]. *Candida albicans* (*C. albicans*) is among the most common fungal commensals and the opportunistic pathogen most frequently identified in fungal infections in humans by virtue of biofilm formation [119,120]. Biofilms of *C. albicans* are also a major contributor to the global AMR burden, it is frequently found in cases of systemic candidiasis in which there is a 40% mortality rate [120,121]. The ability of *C. albicans* to form biofilms, composed of extensively intertwined hyphae cells, is partly due to the exceptional morphological plasticity of this organism to switch between various cell types with distinct morphologies and this has consequences in health and disease [119].

**Figure 6.**
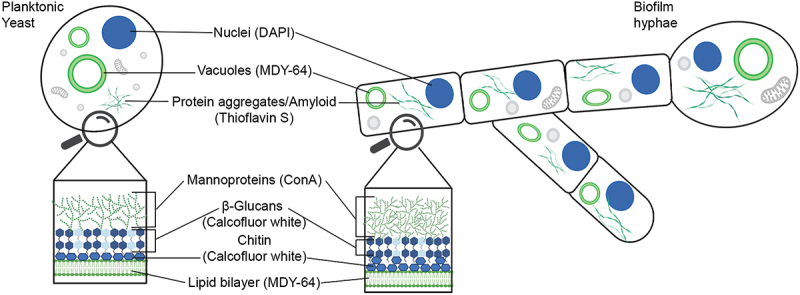
Schematic summary of immunofluorescence analyses of yeast cells (planktonic yeast) and biofilms (biofilm hyphae). The molecular probes (in parenthesis) used to detect cellular structures and organelles as indicated are shown. Reprinted from Kärkkäinen et al. 2022 [122], (open access CC by 4.0).

**Figure 7.**
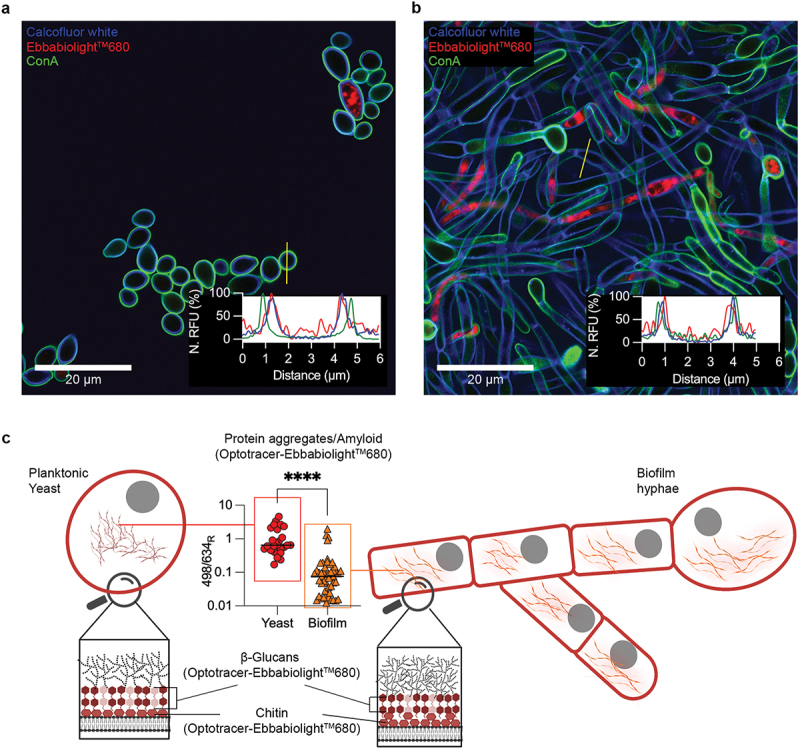
Optotracing analysis of different yeast lifestyles. Optotracing of (a) yeast cells and (b) biofilms using EbbaBiolight^TM^680. Surface attached yeast cells, grown for 72 h in Sabouraud Dextrose Broth supplemented with EbbaBiolight^TM^680, were stained with Calcofluor white (blue) and Concanavalin A (ConA) (green) prior to fluorescence confocal microscopy. Cell walls, to which EbbaBiolight^TM^680 had bound, can be made visible in post-imaging enhancements of the fluorescence micrographs. Embedded in (a) and (b) are line profiles showing the normalized fluorescence intensities (N. RFU) of EbbaBiolight^TM^680 (red), Calcofluor white (blue) and ConA (green) at locations indicated by the yellow lines. (c) Schematic summary showing the cellular structures and organelles in yeast cells and biofilms detected by EbbaBiolight^TM^680. The embedded graph shows the different photophysical properties of EbbaBiolight^TM^680 when bound to intracellular amyloid protein in yeast cells and biofilm. Data represents the ratio of emission intensities at 498 nm and 634 nm (498/634_R_) at 26 regions of interest (ROI) in yeast cells (red circles) and 51 ROI in biofilm (orange triangles) from three independent experiments. The mean of all ROIs is shown, **** denotes P-value < 0.0001. Panels (a, b) are reprinted and panel (c) is adapted from Kärkkäinen et al. 2022 [122], (open access CC by 4.0).

Irrespective of morphology, the main constituents of the cell wall of *C. albicans* are mannoproteins, chitins, and α- and β- linked glucans, whose relative amounts may vary between cell types (Figure 6) [123,124]. Recognizing the similarities of glucans and chitin with cellulose, Kärkkäinen *et al.* analyzed whether the fungal cell wall could act as a target for optotracers, and if so, enable detection of this pathogen [122]. Using purified extracts, productive interactions between the optotracer and commercially available preparations of chitin and β-1,3-glucans were found [122]. Analysis of the optotracer’s photophysical changes revealed the characteristic shift in absorption and emission patterns, alongside the on-switch of fluorescence [122]. While the spectral signature of each individual polysaccharide was close to identical, the optotracer showed a stronger affinity for chitin over β-1,3-glucan when mixes of polysaccharides were used [122]. Interestingly, by adding the optotracer to the growth media, continuous live staining of yeast (Figure 7(a)) and biofilm cells (Figure 7(b)) revealed the presence of large intracellular protein aggregates not previously described [122]. Spectral analysis of the photophysical states of bound optotracers showed distinct differences between yeast and biofilm cells, implying that the nature of the protein aggregates differed between cell types (Figure 7(c)). Taking advantage of this difference, a method for differentiation of *C. albicans* yeast and biofilm cells was developed using spectrophotometry and spectral imaging analysis [122]. Within the cell wall, low-intensity optotracer fluorescence was observed. Co-staining with reporters for mannoproteins, chitins, and β- linked glucans revealed that the optotracer staining colocalized with the latter two components, corroborating results from optotracing analysis of pure compounds (Figure 7(a,b)) [122]. Importantly, the altered staining pattern and presence of protein aggregates in yeast versus hyphenated biofilm cells were not observed when conventional stains for polysaccharide, DNA, and amyloid protein were used. Taken together, this demonstrates optotracing as a useful tool for rapid fungal and biofilm detection tools in research and healthcare.

## 5. Clinical applications of optotracing in infection

### 5.1. Antimicrobial Susceptibility Testing (AST) of biofilm bacteria

While it is recognized that biofilms represent the main form of active bacterial and archaeal life, the vast majority of analytical tools and methods used in research and clinical settings focus on bacteria in a planktonic state of growth [10,125]. The distinction between planktonic and biofilm lifestyles is important since the bacterial physiology differs greatly between the two states. Biofilm cells are encased by the ECM which grants bacteria with protection and increased survivability by providing improved adherent qualities and a secure, spatially organized, and structured environment giving resistive mechanical support. In combination, this makes biofilm-related infections persistent and difficult to eradicate, beyond what current antibiotics are able to deal with [126–128]. It is also becoming increasingly recognized that biofilms play a role in providing bacteria multifactorial resistance towards antibiotics. This involves interference with antibiotic activity, restricted access to bacteria, concealment of abiding sites, desensitization of bacteria, dormancy, or reduced metabolic activity, and extrusion of antibiotics via efflux pumps [129,130]. Collectively, this illustrates that the antibiotic resistance of cells in the biofilm lifestyle is distinct and different from natural or innate resistance mechanisms [131].

**Figure 8.**
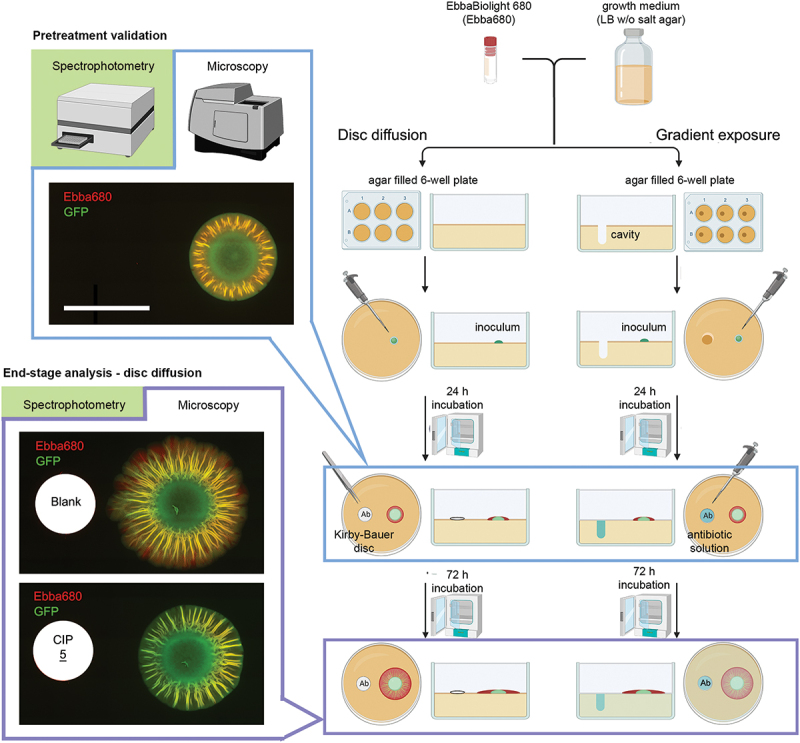
Workflow of optotracer-based biofilm-ASTs. The optotracer EbbaBiolight^TM^680 is added to biofilm-promoting nutrient agar, here LB without salt agar, which is added to the wells of 6-well plates. To perform disc diffusion tests, 10 μl inoculum from a GFP-expressing *Salmonella* culture in exponential phase is placed on top of the agar 10 mm off-center in each well. Following incubation at 28°C for 24 h, formation of pre-treatment biofilms is validated by automated fluorescence microscopy and pre-treatment data is collected. The physical dimensions of the biofilm are measured in phase contrast images, and the amount of ECM-curli is quantified by spectrophotometric recordings. Antibiotic-containing Kirby-Bauer discs are then placed 10 mm from the center of pre-treatment biofilms using blank discs as control. Following incubation at 28°C for 72 h, end-stage analysis of the biofilms is performed as described for the pre-treatment validation. Embedded images show images from automated fluorescence microscopy of mock- (Blank) and ciprofloxacin (CIP)-treated biofilms. To perform gradient exposure for dose-response analysis, a cavity is created in the EbbaBiolight^TM^680-supplemented agar, positioned 10 mm off-center in the 6-well plates. The experimental procedure for pre-treatment and end-stage analysis follows the same process, except that antibiotic exposure is initiated by addition of 80 μl antibiotic in solution to the cavities. Adapted from Eckert et al. 2022 [132], (open access CC by 4.0).

**Figure 9.**
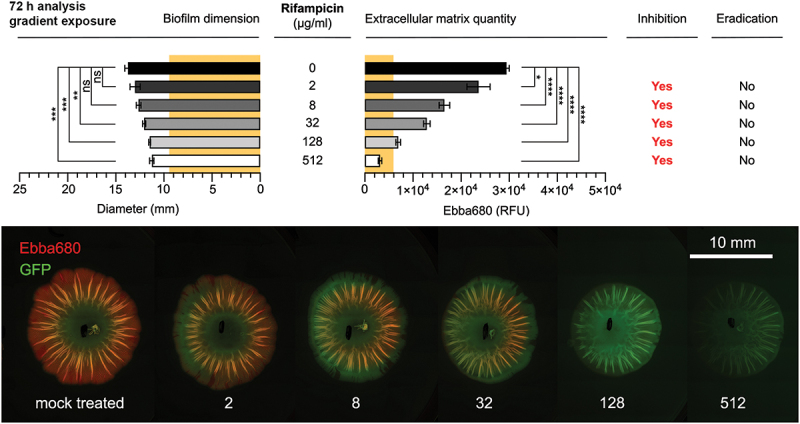
Rifampicin dose-response biofilm-AST of *Salmonella* biofilm. Effects of different concentrations of rifampicin on the diameter (mm) and the quantity of ECM-curli measured by fluorescence (RFU) from EbbaBiolight™680 bound to curli in biofilm formed by the GFP-expressing strain *Salmonella* Enteritidis 3934-p2777. The diameter (9.5 mm) and quantity of ECM-curli (5926 RFU) in the pre-treatment biofilm is indicated in the graphs by the yellow background. Inhibitory or eradication effects of rifampicin on the biofilms are indicated by yes/No. Data is based on three experimental replicates with two technical repeats. P-values <0.05, <0.01, <0.001 and < 0.0001 are indicated as *, **, *** and ****, ns = non-significant. The fluorescence images in the bottom panel visualize the effects of different concentrations of rifampicin (0–512 μg/ml) on biofilm formation. Images were collected by automated microscopy Reprinted from Eckert et al. 2022 [132], (open access CC by 4.0).

In light of the global AMR crisis, the success of future antibiotic development will depend on novel AST methods that assess the effects of existing and prospective new compounds on bacteria in the biofilm lifestyle. Pioneering biofilm-targeted diagnostics and – therapies, an optotracer-based methodology has been developed in which the effect of antibiotics on biofilms and biofilm cells can be quantified and visualized (Figure 8) [132]. Improving on prevailing biofilm assays that are conventionally performed in Petri dishes, the quantitative morphotypical optotracer assay was housed in a 6-well plate, in order to make it compatible with automation [114,133]. By adding optotracers to the nutrient agar, the effect of various commercially available antibiotics on the ECM production and the physical size of the macrocolony could be analyzed by fluorescence microscopy and spectroscopy [132]. This method identified several of the antibiotics’ biofilm-relevant effects, defined as biofilm-static, biofilm-cidal, and biofilm dispersal, all reflecting the biofilm lifestyle of bacteria [134]. Unique to the optotracer-based biofilm-AST is that it allows biofilm to form *prior* to antibiotic exposure [132]. Validation of biofilm formation and recording of biofilm parameters (dimension, morphology, amount of ECM) prior to antibiotic exposure are essential to analyze whether an antibiotic is active against biofilm bacteria. Based on the fluorescence signals from optotracers bound to the curli component of the ECM (ECM-curli), these parameters were first determined in a *Salmonella* biofilm formed on the agar of the 6-well plate after 24 h incubation, before any antibiotics were applied [132]. Following application of antibiotic-containing Kirby-Bauer discs, or antibiotics in solution administered in a small well in the agar in close vicinity to the biofilm, incubation of the 6-well plate was continued for 72 h. By recording optotracer fluorescence at the end-stage, inhibition of the biofilm, defined by reduction of the quantity of ECM-curli, identified the antibiotic compounds most effective towards biofilms. Importantly, the method also revealed several novel biofilm-specific effects of the tested compounds [132]. For example, treatment of biofilms for 72 h with high concentration of rifampicin resulted in biofilms with significantly lower ECM-curli quantities as compared to the non-exposed biofilm that had been grown for 24 h prior to exposure (Figure 9) [132]. The optotracer-based biofilm-AST thus presents as a novel method, fit to renew our understanding and expectations of the efficacy of current antimicrobial compounds on biofilm infections. Also, it has great potential serving as a future tool for diagnostics of biofilm infections, and for the development of biofilm-specific antibiotics. Taken together, the biofilm-AST is expected to improve the treatment outcome of biofilm infections.

### 5.2. Diagnosing biofilm infections

Many biofilm-forming bacterial species produces polysaccharides as major constituents of their ECM. Cellulose is in addition to *Salmonella* and *E. coli*, synthesized by numerous bacteria, such as *Mycobacterium tuberculosis* and *Acetobacter xylinum* [135,136]. Along the microbial production of cellulose, this polysaccharide is synthesized by members of the Plantae kingdom and is therefore considered the most abundant organic compound on Earth [137]. In contrast, members of the Animalia kingdom, which includes humans and mammals, do not produce cellulose. Taking advantage of this distinction, Antypas et al., investigated a diagnostic application of optotracing, in which the spectra were used to identify bacterially produced cellulose in urine as a marker of biofilm-associated urinary tract infection (UTI) [113]. Representing the first non-disruptive method for cellulose detection, optotracing-based biofilm diagnostics in UTI is of great importance, given that 150 million people suffer from UTI annually, and that the biofilm lifestyle is linked to recurrent and persistent infections and to the development of antimicrobial resistance [138,139].

**Figure 10.**
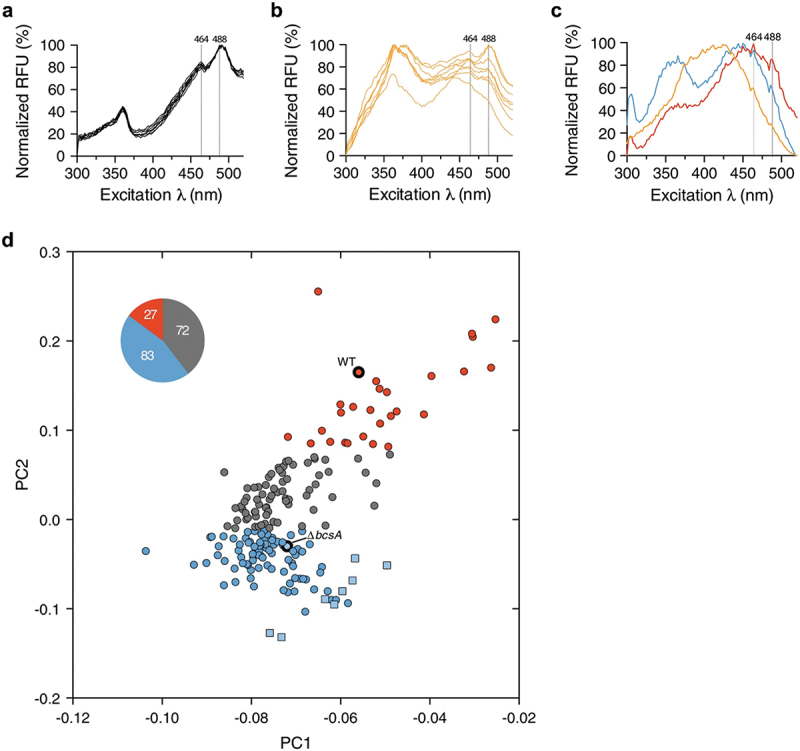
Optotracing diagnostics of biofilm infection in the urinary tract. (a) optotracing for direct detection of cellulose in PBS. The fluorescence signals from the optotracer, excited at 300–520 nm with emission collected at 545 nm, are shown in a normalized excitation spectra. Solid lines show average fluorescence of two technical replicates, dotted vertical lines at 464 and 488 nm represent the cellulose signature. (b) normalized excitation spectra showing optotracing for cellulose in urine collected from eight healthy volunteers. The urine was spiked with microcrystalline cellulose, and mixed with the optotracer. Optotracer fluorescence are shown in a normalized excitation spectrum. Lines show the average fluorescence of three technical replicates. (c) normalized excitation spectra of fluorescence from optotracers bound to biofilms formed by the uropathogenic strains UPEC 12, (wt) (red) and isogenic mutant *ΔbcsA* (blue). The biofilms were recovered from urine samples that had been spiked with the strains. As optotracing of recovered biofilms were performed in PBS, a spectrum of optotracer in PBS (orange) is included for comparison. Average normalized fluorescence from three technical replicates from one representative experiment is shown. (d) optotracing analysis of the presence of cellulose in urine from patients with confirmed UTI. The study includes 182 urine samples from UTI patients (circle), 8 urine samples from healthy volunteers (square), and biofilm preparations of UPEC 12 (wt) and *ΔbcsA* for comparison. Spectral data in the range 464–508 nm (representing the cellulose signature) of the normalized excitation spectra were analyzed with Principal component analysis (PCA) and clustered with ‘k means clustering’. The three generated clusters differentiate samples that are positive (red circles, clustering with thick border red circle of UPEC12 wt) and negative (blue circles, clustering with thick border blue circle of UPEC 12 *ΔbcsA*).) for cellulose, as well as samples with insufficient discriminatory performance (gray). The pie chart inset shows the number of UTI samples in each of the three clusters. Reprinted from Antypas et al. 2018 [113], (open access CC by 4.0).

Uropathogenic *E. coli* (UPEC) is the main causative pathogen of UTI. Using microcrystalline cellulose sourced from plants as comparator, spectrophotometric recordings of absorption and emission spectra of optotracers bound to cellulose in the ECM of a clinical UPEC isolate were used to identify a cellulose-specific optical signature, which next was used to identify cellulose in the urine samples (Figure 10(a–c)) [113]. Importantly, the optical signature of UPEC cellulose was consistent with earlier *Salmonella* biofilm studies, as well as work performed in parallel looking into the minimal stereochemical requirements for optotracers to bind to cellulose and other glucan-based polysaccharides [90,140]. Extending the analysis from lab conditions to a clinical microbiology setting, urine samples from 182 patients diagnosed with UTI were screened, using healthy volunteers as control. Following the addition of the optotracer to the clinical samples, spectrophotometric recordings were performed to probe for the optical signatures of ECM-cellulose [113]. To circumvent the difficulty to analyze optical spectra by eye, a data processing workflow was developed that enabled automatic comparison of optotracer signals from clinical urine samples and control samples based on genetically well-characterized strains unable to produce ECM-cellulose [113]. Utilizing principal component analysis and k-means clustering, this workflow categorized 27 urine samples as positive for cellulose (Figure 10(d)) [113]. From this, it was concluded that a minimum of 15% of UTI patients suffer from a biofilm-associated UTI infection. Translating this to the global scale, 22.5 million patients would suffer from biofilm-UTI annually.

This result is significant for several reasons: *i)* it represents a culture-independent alternative by which UTI is diagnosed based on cellulose as a bacterial biomarker; *ii)* by detecting ECM-cellulose in a patient’s urine samples, direct evidence is provided of a biofilm infection in the urinary tract [113]. This study represents the first example of direct diagnostics of biofilm-associated infections, made possible only by the non-disruptive ability of optotracing to detect polysaccharides in their native state [113]. Accordingly, this study has laid the foundation for optotracing in creating new bedside diagnostic tools, thereby improving patient care and guide the development of biofilm-effective treatment strategies.

**Figure 11.**
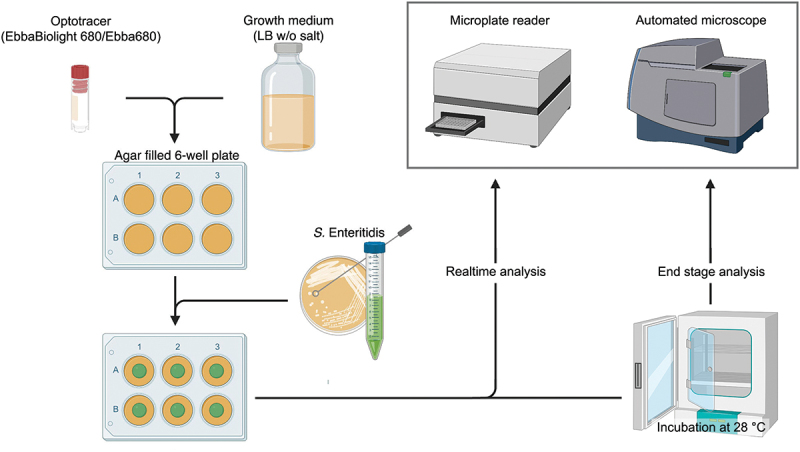
Workflow for semi-high throughput screening on biofilm formation. Wells of a six well-plate are filled with 2 ml LB agar without salt, supplemented with the optotracer EbbaBiolight™680. The bacterial inoculum is placed on the agar center. The plate is incubated in different instruments depending on the experiment. *End stage recording*: After incubation in a standard incubator, fluorescence signals from the biofilms are analyzed in a microplate reader or an automated microscope. *Real-time recording*: Dynamics of biofilm formation is studied by incubating the plate in a temperature-controlled microplate reader, programmed to record spectra at defined time intervals. Alternatively, the plate is incubated in a temperature-controlled automated microscope that provides a cinematic view of biofilm formation over ca 70 h. Reprinted from Choong et al. 2021 [114], (open access CC by 4.0).

**Figure 12.**
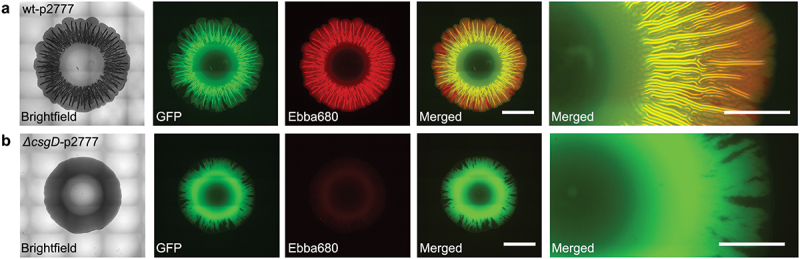
Automated microscopy showing biofilms formed in the 6-well plate optotracing method, in which bacteria grow on agar supplemented with the optotracer EbbaBiolight™680 (Ebba680). Morphologies of the GFP-expressing *Salmonella* strains (a) wt-p2777 (curli+, cellulose +) and (b) *ΔcsgD*-p2777 (curli-, cellulose-) are shown in brightfield images, while fluorescence microscopy shows the spatial distribution of bacteria (GFP, green) and ECM-curli (Ebba680, red) separately and in merged images of respective strain. Scale bar = 5 mm. Enlarged sections from the merged images of each strain are shown to the right. Scale bar = 2 mm. Adapted from Choong et al. 2021 [114], (open access CC by 4.0).

An important aspect is the need of high-throughput screening methodologies and automated data analyzes to provide clear and impartial information vital for infection diagnosis and treatment. This need is particularly critical in categorizing biofilm producing strains among the clinical isolates. To find solutions for this purpose, an application of optotracers was described when a semi-high throughput method for screening of microbial samples for biofilm formation (Figure 11) was presented [114]. Improving on conventional morphotypical methods housed in Petri dishes, such as the Congo red and Calcofluor white-based assays, a multi-well plate format was used as a means to simultaneously analyze multiple samples [114]. This format took advantage of the facts that optotracers are non-toxic to bacteria and can therefore be present in the growth media, and that optotracers gives fluorescent signals only when they bind to a target. Collectively, this gives optotracers the unique feature of acting as continuous reporters, providing kinetic information of curli expressing biofilms when added as a supplement to nutrient agar [114]. Comparisons of biofilm developed by bacteria growing on optotracer-containing agar casted in Petri dishes and 6-well plates revealed morphological consistency in both vessels. Also, a reproducible uniformity of the physical dimension was observed of biofilms in the 6-well plate [114]. Using a microplate reader or automated microscope, the kinetic appearance of fluorescence from optotracers binding to ECM-curli was recorded, thereby translating the qualitative-based assessments used in conventional biofilm assays into a quantitative, binary readout (Figure 12(a,b)). The method represents a notable advancement to the field of biofilm detection in both laboratory and clinical settings by providing results less open to interpretation and experience biases, while increasing the throughput and efficiency of experimentation.

## 6. Iterating between bacteria and plants to understand infections

Current medical knowledge focuses mostly on the role of proteins in health and disease. This has been facilitated by the ubiquitous use of antibodies for detection, imaging, and analysis. The roles of polysaccharides are somewhat ignored, partly due to a lack of analytical techniques. Guided by the need to understand the role of bacterial biofilm in infection and antibiotic resistance, optotracing has been developed as a ground-breaking molecular tool for polysaccharide analytics. Interestingly, many polysaccharides produced by bacteria to give structural support to the biofilm communities can also be found in plant tissues. Current knowledge and predictability of polysaccharide composition in plant biomass is substantially more advanced than in bacterial biofilms. By combining different competences, development of the optotracing technology has been iterating between the two fields. This has generated new tools and knowledge in the biofilm research field, while at the same time offered important new technologies to the plant science field in general, and biomass extraction and utilization of biopolymers in particular. Although not central to theme of the current review, the parallel development of technology in the fields of bacterial infection and plant science is a nice illustration of unexpected outputs that only happens in openminded interdisciplinary research settings.

**Figure 13.**
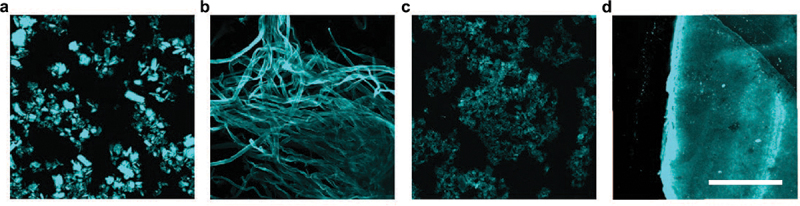
Optotracing for cellulose detection. Fluorescence confocal imaging of (a) microcrystalline cellulose, (b) pulp cellulose, (c) cellulose nanofibrils, and (d) paper made of cellulose nanofibrils, stained with an optotracer. Excitation at 473 nm and bandwidth filters detecting 490–530 nm were applied. Scale bar = 200 μm. Reprinted from Choong et al. 2016 [89], (open access CC by 4.0).

**Figure 14.**
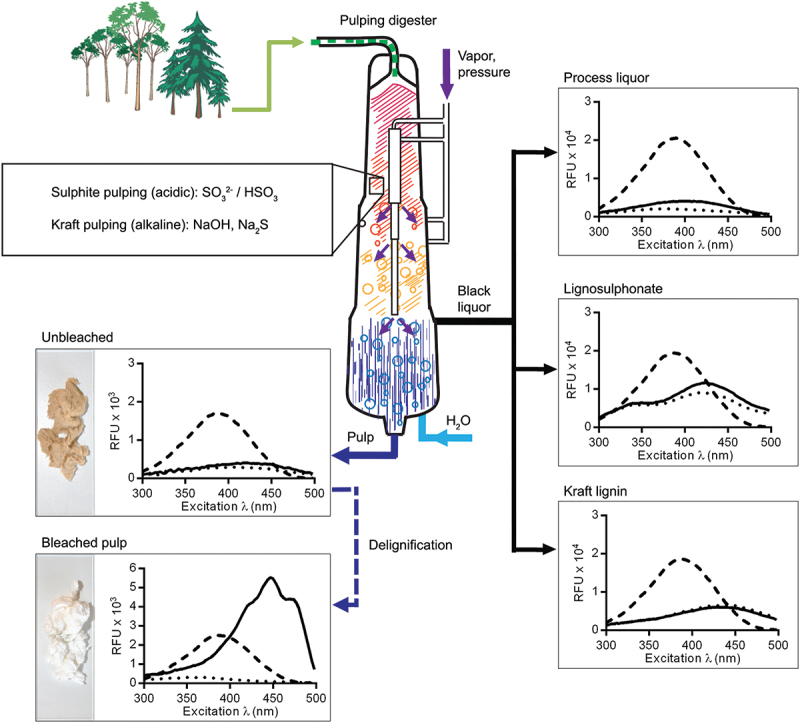
Cartoon illustrating the continuous cooking process, presenting key stages for quality analysis of cellulose and by-products. Graphs show the excitation spectra of process liquor, lignosulphonate, Kraft lignin, unbleached pulp, and bleached pulp with added optotracer (solid lines), compared to spectra of the optotracer alone (dashed lines), as well as the basal fluorescence of each sample (dotted lines). Photographs show the characteristic colors of unbleached (brownish) and bleached (white) pulp. Reprinted from Choong et al. 2016 [89], (open access CC by 4.0).

Parallel to the development of optotracing in biofilm detection, the applicability of optotracers to detect cellulose, lignin, and hemicelluloses, the three main lignocellulosic components of biomass derived from plants, was evaluated [89]. Binding of a pentameric optotracer to microcrystalline cellulose resulted in a unique pattern of absorption and emission that included the on-switch of fluorescence, referred to in subsequent studies as the optical signature of cellulose [89]. The increase in optotracer fluorescence was directly proportional to the concentration of cellulose. Continuous measurements of microcrystalline cellulose and cellulose nanofibrils in the presence of the cellulose-degrading enzyme cellulase showed a decrease of optotracer fluorescence over time, making the enzymatic reaction clearly visible and quantifiable in real-time [89]. In contrast, binding of the optotracer to lignin resulted in no detectable fluorescence, likely due to the physicochemical process of fluorescence quenching by this complex plant polymer [141]. Lignin refers to a diverse group of heterogeneous polymers formed by the combination of a number of lignols crosslinked at a variety of different positions [142]. Probing this interaction by optotracing, an inverse but linear relationship was found between the concentration of lignin and the loss of signal [89]. Albeit indirect, the ability of optotracers to detect lignin is significant owing to the great potential of this renewable precursor in a variety of products, such as biofuels, plastics, and additives [143]. Like lignin, hemicellulose is a highly useful renewable material with numerous uses. Hemicelluloses represent a mixed group of polymers made from C5 and C6 sugars, such as xylose, arabinose, mannose, and galactose [144,145]. Probing the interactions between optotracers and representatives of different types of hemicelluloses, an inverse relationship between the concentration of each polysaccharide and the tracer’s fluorescence was observed, implying that the tracking of these polymers was possible [89]. Focusing on the detection of cellulose, multiple scenarios were analyzed in which optotracing could be applied. Comparative analysis of cellulose morphologies, represented by pulp cellulose, microcrystalline cellulose, cellulose nanofibrils, and paper made from nanocellulose, all showed the characteristic optical signature of optotracers bound to cellulose [89]. Fluorescence microscopy analysis of optotracer-stained cellulose enabled visual assessment of the morphologies, comparable to the conventional method of scanning electron microscopy (Figure 13(a-d)). Expanding the analysis to lignin-rich liquors, brown pulp, and bleached pulp sampled from the pulp extraction process revealed a high sensitivity of optotracers for the presence of lignin and the potential use as a highly sensitive indicator of cellulose purity and the effectiveness of the extraction process (Figure 14) [89].

**Figure 15.**
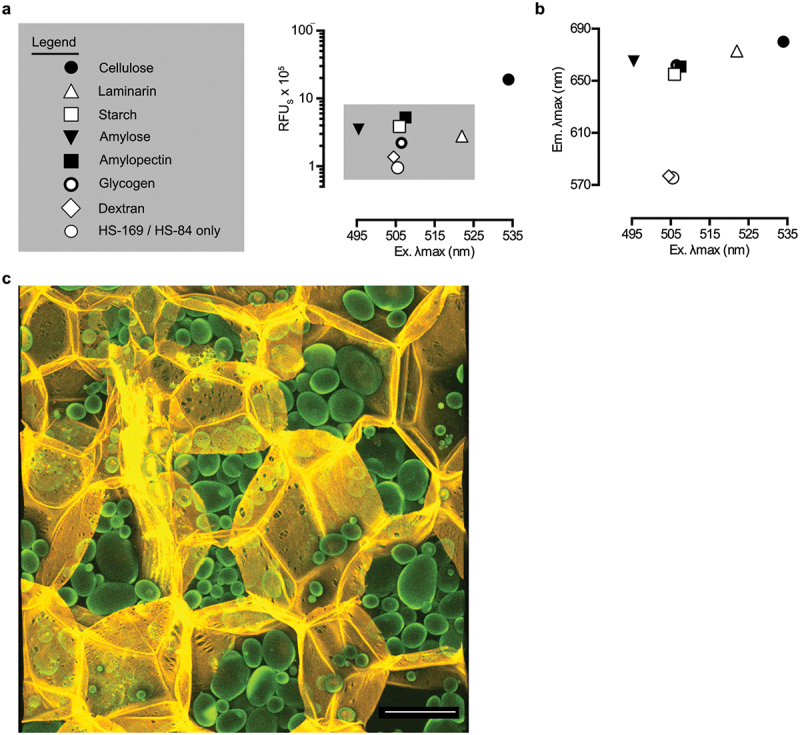
EbbaBiolight™680 enables stereochemical differentiation of glycosidic linkages in glucans. (a) optical signatures, defined by plotting emitted fluorescence intensities (RFU) against λ_max_ from the excitation spectra (Ex. λ_max_), of Carbotrace™680 interacting with microcrystalline cellulose, laminarin, starch, amylose, amylopectin, glycogen and dextran. Each data point represents the average from three independent experiments. (b) optical signatures, defined by plotting λ_max_ from the emission spectra (Em. λ_max_) against Ex. λ_max_ for experiments shown in (a), reveal stereochemistry-dependent clustering of glucans. (c) the non-disruptive optotracing technique enables carbohydrate anatomical mapping in plant tissues. Multi-laser/multi-detector analysis of a thin slice of potato stained by Carbotrace™680 shows that cellulose (pseudo-colored in yellow) locates to the cell walls of the large, multi-faced parenchyma cells, where structural features such as plasmodesmata and small intercellular pores are clearly observed. Starch (pseudo-colored in green) locates to intracellular granules, whose size and number per cell varies greatly. The contrast between a strongly fluorescent periphery and less intense inner core of the starch granules indicates incomplete penetration of Carbotrace™680 to the dense inner compartments. The micrograph represents a 3D brightest point projection of an image stack (25.52 μm stack, z-step = 1.16 μm) collected by confocal microscopy. Scale bar = 100 μm. Reprinted from Choong et al. 2019 [100], (open access CC by 4.0).

When probing the sensitivity of optotracers for identifying the stereochemical structure of glucans, a remarkable resolution was revealed. Intricate details in the absorption and emission patterns of bound optotracers differentiated the α and β configured glucopyranosyl units in glucans [140]. Further analysis of modified cellulose showed an ability of optotracers to detect the presence and the identity of chemical additions to the cellulose backbone [140]. By introducing a donor– acceptor–donor type electronic structure to the optotracer, the resolution was further improved, allowing α(1–2), α(1–4), α(1–6), β(1–3), and β(1–4) linked glucans to be differentiated at the molecular level (Figure 15(a,b)) [100]. The non-destructive optotracing technique was instrumental in a study by Wahlström *et al.*, where it was applied to differentiate glucans from xyloglucans [146].

Taking advantage of the newfound ability to detect multiple materials, optotracing was further developed as a fluorescence microscopy-based method to visually assess the composition of glucans and their histological location in plant tissues. Using optotracing of potato tissue as example, an anatomical map was generated in which the fluorescence from the optotracer bound to cellulose outlined the cell walls with exceptional details, whereas binding to starch revealed that this macromolecule is located in dense intracellular starch granules (Figure 15(c)). By mapping the location of carbohydrates in plant tissues, optotracing can greatly contribute to a circular bioeconomy by generating a holistic understanding of the compartmentalized composition of a biomass without the exhaustive trial and error process of the convention to date.

## 7. Conclusions and future perspective

Our world faces numerous challenges, some directly related to health issues, others to responsible use of resources. No matter the field, sensors are needed to analyze and control processes. We believe that development of direct sensing technologies is essential to improve future diagnostics, saving valuable time, while accurately identify the optimal therapy. Several research centers around the world are specifically focusing on understanding the biofilm lifestyle of bacteria. By combining the massive output of biofilm research with sensor development and material sciences, we foresee the development of new approaches not only to detect and diagnose infections, but also for the generation of biofilm-specific antibiotics. Collectively, these efforts are needed to empower us, giving us means to counteract the threat posed by antimicrobial resistance development.

While maybe not obvious at the first glance, infection and plant science have a lot in common. The rigid bacterial cell wall and the structural support in bacterial biofilms rely on polysaccharides, which are the same macromolecules as in plants. With research becoming increasingly interdisciplinary, merger of competences and developments from different fields will act synergistically to improve our knowledge in multiple fields. That is why infection research can deliver a cutting-edge technology to the plant science field, in which optotracing represents the first method for non-destructive analysis of polysaccharides, thereby allowing their analysis in native states. Bioeconomy broadly means the production and use of biological resources, products, and processes in such ways that fossil resources can be replaced. Key to achieving the goal of circular bioeconomy is a responsible use of natural resources [147]. In contrast to current sample destructive methods, the non-destructive optotracing method serves as an enabling technology for development towards efficient use of plant-derived materials and biomass [100,140]. By finding applications in both infection and production of fossil-free materials, optotracing is an illustrative example of the power of interdisciplinary research, helping us to find solutions to the major global challenges we are facing today.

## References

[1] Hecker MT, Aron DC, Patel NP, et al. Unnecessary use of antimicrobials in hospitalized patients: current patterns of misuse with an emphasis on the antianaerobic spectrum of activity. Arch Intern Med. 2003;163(8):972–24. doi: 10.1001/archinte.163.8.972 12719208

[2] Fridkin S, Baggs J, Fagan R, et al. Vital signs: improving antibiotic use among hospitalized patients. Morb Mortal Wkly Rep. 2014;63:194–200. PMC4584728 24598596

[3] Vaughn VM, Flanders SA, Snyder A, et al. Excess antibiotic treatment duration and adverse events in patients hospitalized with pneumonia: a multihospital cohort study. Ann Intern Med. 2019;171:153–163. doi: 10.7326/M18-3640 31284301 PMC13258130

[4] Krockow EM, Kurvers RHJM, Herzog SM, et al. Harnessing the wisdom of crowds can improve guideline compliance of antibiotic prescribers and support antimicrobial stewardship. Sci Rep. 2020;10:18782. doi: 10.1038/s41598-020-75063-z 33139823 PMC7608639

[5] Landstedt K, Sharma A, Johansson F, et al. Antibiotic prescriptions for inpatients having non-bacterial diagnosis at medicine departments of two private sector hospitals in Madhya Pradesh, India: a cross-sectional study. BMJ Open. 2017;7(4):e012974. doi: 10.1136/bmjopen-2016-012974 PMC5775474 28391232

[6] Rao GG. Risk factors for the spread of antibiotic-resistant bacteria. Drugs. 1998;55:323–330. doi: 10.2165/00003495-199855030-00001 9530540

[7] Aldred KJ, Kerns RJ, Osheroff N. Mechanism of quinolone action and resistance. Biochemistry. 2014;53(10):1565–1574. doi: 10.1021/bi5000564 24576155 PMC3985860

[8] Mah T-F. Biofilm-specific antibiotic resistance. Future Microbiol. 2012;7(9):1061–1072. doi: 10.2217/fmb.12.76 22953707

[9] Sharma D, Misba L, Khan AU. Antibiotics versus biofilm: an emerging battleground in microbial communities. Antimicrob Resist Infect Control. 2019;8:76. doi: 10.1186/s13756-019-0533-3 31131107 PMC6524306

[10] Bowler P, Murphy C, Wolcott R. Biofilm exacerbates antibiotic resistance: is this a current oversight in antimicrobial stewardship? Antimicrob Resist Infect Control. 2020;9(1):162. doi: 10.1186/s13756-020-00830-6 33081846 PMC7576703

[11] Murray CJL, Ikuta KS, Sharara F, et al. Global burden of bacterial antimicrobial resistance in 2019: a systematic analysis. Lancet. 2022;399:629–655. doi: 10.1016/S0140-6736(21)02724-0 35065702 PMC8841637

[12] O’Neill J. 2018. Tackling drug-resistant infections globally: final report and recommendations. 2016

[13] World Bank and World Health Organization. Sustaining action against antimicrobial resistance: a case series of country experiences. Washington DC, USA: World Bank; 2022.

[14] Lavigne J-P, Espinal P, Dunyach-Remy C, et al. Mass spectrometry: a revolution in clinical microbiology? Clin Chem Lab Med. 2013;51(2):257–270. doi: 10.1515/cclm-2012-0291 23072853

[15] Preiswerk B, Imkamp F, Vorburger D, et al. Mycoplasma penetrans bacteremia in an immunocompromised patient detected by metagenomic sequencing: a case report. BMC Infect Dis. 2020;20(7). doi: 10.1186/s12879-019-4723-7 PMC6942334 31900105

[16] Philipp Dellinger R, Carlet J. Sepsis handbook: early diagnosis of Sepsis. BioMérieux, Lyon, France. 2007.

[17] Chan T, Gu F. Early diagnosis of sepsis using serum biomarkers. Expert Rev Mol Diagn. 2011;11(5):487–496. doi: 10.1586/erm.11.26 21707457

[18] The Authors BJU International. NICE (2017) Sepsis: recognition, diagnosis and early management. BJU Int. 2018;121:497–514. doi: 10.1111/bju.14179 29603898

[19] Zumla A, Al-Tawfiq JA, Enne VI, et al. Rapid point of care diagnostic tests for viral and bacterial respiratory tract infections–needs, advances, and future prospects. Lancet Infect Dis. 2014;14:1123–1135. doi: 10.1016/S1473-3099(14)70827-8 25189349 PMC7106435

[20] Mahony JB, Petrich A, Smieja M. Molecular diagnosis of respiratory virus infections. Crit Rev Clin Lab Sci. 2011;48:217–249. doi: 10.3109/10408363.2011.640976 22185616

[21] Tenover FC. Developing molecular amplification methods for rapid diagnosis of respiratory tract infections caused by bacterial pathogens. ClinInfect Dis. 2011;52(Suppl 4):S338–45. doi: 10.1093/cid/cir049 21460293

[22] Alexander DJ, Brown IH. Recent zoonoses caused by influenza A viruses. Revue Scientifique et Technique de l’OIE. 2000;19(1):197–225. doi: 10.20506/rst.19.1.1220 11189716

[23] Al-Ahmadi K, Alahmadi M, Al-Zahrani A. Spatial association between primary Middle East respiratory syndrome coronavirus infection and exposure to dromedary camels in Saudi Arabia. Zoonoses Public Health. 2020;67(4):382–390. doi: 10.1111/zph.12697 32112508 PMC7228245

[24] Van Reeth K. Avian and swine influenza viruses: our current understanding of the zoonotic risk. Vet Res. 2007;38(2):243–260. doi: 10.1051/vetres:2006062 17257572

[25] Anon WHO Coronavirus (COVID-19) Dashboard.

[26] Brezmes MF, Ochoa C, Eiros JM. Cost analysis in a clinical microbiology laboratory. Eur J Clin Microbiol Infect Dis. 2002;21:582–588. doi: 10.1007/s10096-002-0776-3 12226688

[27] Cantón R, Gómez G, de la Pedrosa E. Economic impact of rapid diagnostic methods in clinical microbiology: price of the test or overall clinical impact. Enferm Infecc Microbiol Clin. 2017;35:659–666. doi: 10.1016/j.eimc.2017.09.005 29033026

[28] Thomson RB Jr. One small step for the gram stain, one giant leap for clinical microbiology. J Clin Microbiol. 2016;54:1416–1417. doi: 10.1128/JCM.00303-16 27008876 PMC4879305

[29] Kralik P, Ricchi M. A basic guide to real time PCR in microbial diagnostics: definitions, parameters, and everything. Front Microbiol. 2017;8:108. doi: 10.3389/fmicb.2017.00108 28210243 PMC5288344

[30] Frickmann H, Zautner AE, Moter A, et al. Fluorescence in situ hybridization (FISH) in the microbiological diagnostic routine laboratory: a review. Crit Rev Microbiol. 2017;43(3):263–293. doi: 10.3109/1040841X.2016.1169990 28129707

[31] Zourob M, Elwary S, Turner APF. Principles of bacterial detection: biosensors, recognition receptors and microsystems. Springer Science & Business Media; 2008. doi: 10.1007/978-0-387-75113-9

[32] Li L, Mendis N, Trigui H, et al. The importance of the viable but non-culturable state in human bacterial pathogens. Front Microbiol. 2014;5. doi: 10.3389/fmicb.2014.00258 PMC4040921 24917854

[33] Reller LB, Weinstein M, Jorgensen JH, et al. Antimicrobial susceptibility testing: a review of general principles and contemporary practices. Clin Infect Dis. 2009;49:1749–1755. doi: 10.1086/647952 19857164

[34] Leclercq R, Cantón R, Brown DFJ, et al. EUCAST expert rules in antimicrobial susceptibility testing. Clin Microbiol Infect. 2013;19(2):141–160. doi: 10.1111/j.1469-0691.2011.03703.x 22117544

[35] Kerremans JJ, Verboom P, Stijnen T, et al. Rapid identification and antimicrobial susceptibility testing reduce antibiotic use and accelerate pathogen-directed antibiotic use. J Antimicrob Chemother. 2008;61:428–435. doi: 10.1093/jac/dkm497 18156278

[36] MacVane SH, Oppermann N, Humphries RM. Time to result for pathogen identification and antimicrobial susceptibility testing of bronchoalveolar lavage and endotracheal aspirate specimens in U.S. acute care hospitals. J Clin Microbiol. 2020;58. doi: 10.1128/JCM.01468-20 PMC7587099 32878953

[37] Chun K, Syndergaard C, Damas C, et al. Sepsis pathogen identification. J Lab Autom. 2015;20:539–561. doi: 10.1177/2211068214567345 25631157

[38] Maurer FP, Christner M, Hentschke M, et al. Advances in rapid identification and susceptibility testing of bacteria in the clinical microbiology laboratory: implications for patient care and antimicrobial stewardship programs. Infect Dis Rep. 2017;9:6839. doi: 10.4081/idr.2017.6839 28458798 PMC5391540

[39] Wiegand I, Hilpert K, Hancock REW. Agar and broth dilution methods to determine the minimal inhibitory concentration (MIC) of antimicrobial substances. Nat Protoc. 2008;3:163–175. doi: 10.1038/nprot.2007.521 18274517

[40] Hudzicki J. Kirby-Bauer disk diffusion susceptibility test protocol. 2009.

[41] Behera B, Anil Vishnu GK, Chatterjee S, et al. Emerging technologies for antibiotic susceptibility testing. Biosens Bioelectron. 2019;142:111552. doi: 10.1016/j.bios.2019.111552 31421358

[42] Jorgensen JH, Ferraro MJ. Antimicrobial susceptibility testing: general principles and contemporary practices. Clin Infect Dis. 1998;26(4):973–980. doi: 10.1086/513938 9564485

[43] Stres B, Kronegger L. Shift in the paradigm towards next-generation microbiology. FEMS Microbiol Lett. 2019;366. doi: 10.1093/femsle/fnz159 PMC6759065 31314103

[44] Fournier P-E, Drancourt M, Colson P, et al. Modern clinical microbiology: new challenges and solutions. Nat Rev Microbiol. 2013;11(8):574–585. doi: 10.1038/nrmicro3068 24020074 PMC7097238

[45] Freiwald A, Sauer S. Phylogenetic classification and identification of bacteria by mass spectrometry. Nat Protoc. 2009;4(5):732–742. doi: 10.1038/nprot.2009.37 19390529

[46] Welker M, van Belkum A. One System for all: is mass spectrometry a future alternative for conventional antibiotic susceptibility testing? Front Microbiol. 2019;10:2711. doi: 10.3389/fmicb.2019.02711 31849870 PMC6901965

[47] Wieser A, Schubert S. MALDI-TOF MS entering the microbiological diagnostic laboratory – from fast identification to resistance testing. Trends Analyt Chem. 2016;84:80–87. doi: 10.1016/j.trac.2016.05.013

[48] Staley C, Unno T, Gould TJ, et al. Application of Illumina next-generation sequencing to characterize the bacterial community of the Upper Mississippi River. J Appl Microbiol. 2013;115(5):1147–1158. doi: 10.1111/jam.12323 23924231

[49] Westblade LF, van Belkum A, Grundhoff A, et al. Role of clinicogenomics in Infectious disease diagnostics and public health microbiology. J Clin Microbiol. 2016;54:1686–1693. doi: 10.1128/JCM.02664-15 26912755 PMC4922100

[50] Yohe S, Thyagarajan B. Review of clinical next-generation sequencing. Arch Pathol Lab Med. 2017;141(11):1544–1557. doi: 10.5858/arpa.2016-0501-RA 28782984

[51] Onsongo G, Erdmann J, Spears MD, et al. Implementation of cloud based next generation sequencing data analysis in a clinical laboratory. BMC Res Notes. 2014;7:314. doi: 10.1186/1756-0500-7-314 24885806 PMC4036707

[52] Law JW-F, Ab Mutalib N-S, Chan K-G, et al. Rapid methods for the detection of foodborne bacterial pathogens: principles, applications, advantages and limitations. Front Microbiol. 2014;5:770. doi: 10.3389/fmicb.2014.00770 25628612 PMC4290631

[53] Bhakta SA, Evans E, Benavidez TE, et al. Protein adsorption onto nanomaterials for the development of biosensors and analytical devices: a review. Anal Chim Acta. 2015;872:7–25. doi: 10.1016/j.aca.2014.10.031 25892065 PMC4405630

[54] Crivianu-Gaita V, Thompson M. Aptamers, antibody scFv, and antibody Fab’ fragments: an overview and comparison of three of the most versatile biosensor biorecognition elements. Biosens Bioelectron. 2016;85:32–45. doi: 10.1016/j.bios.2016.04.091 27155114

[55] Idil N, Hedström M, Denizli A, et al. Whole cell based microcontact imprinted capacitive biosensor for the detection of Escherichia coli. Biosens Bioelectron. 2017;87:807–815. doi: 10.1016/j.bios.2016.08.096 27657842

[56] Pappa A-M, Parlak O, Scheiblin G, et al. Organic electronics for point-of-care metabolite monitoring. Trends Biotechnol. 2018;36(1):45–59. doi: 10.1016/j.tibtech.2017.10.022 29196057

[57] Parlak O, Richter-Dahlfors A. Bacterial sensing and biofilm monitoring for infection diagnostics. Macromol Biosci. 2020;20:e2000129. doi: 10.1002/mabi.202000129 32588553

[58] Butina K, Löffler S, Rhen M, et al. Electrochemical sensing of bacteria via secreted redox active compounds using conducting polymers. Sens Actuators B. 2019;297:126703. doi: 10.1016/j.snb.2019.126703

[59] Butina K, Filipović F, and Richter-Dahlfors A, et al. 2021. An organic electrochemical transistor to monitor *Salmonella* growth in real‐time. Adv Mater Interfaces. 8:2100961. doi: 10.1002/admi.202100961

[60] Löffler S, Antypas H, Choong FX, et al. Conjugated Oligo- and polymers for bacterial sensing. Front Chem. 2019;7:265. doi: 10.3389/fchem.2019.00265 31058140 PMC6482434

[61] Björk L, Klingstedt T, Nilsson KPR. Thiophene-based ligands: design, synthesis and their utilization for optical assignment of polymorphic-disease-associated protein aggregates. Chembiochem. 2023;24:e202300044. doi: 10.1002/cbic.202300044 36891883 PMC10404026

[62] McQuade DT, Pullen AE, Swager TM. Conjugated polymer-based chemical sensors. Chem Rev. 2000;100(7):2537–2574. doi: 10.1021/cr9801014 11749295

[63] Butina K, Lantz L, Choong FX, et al. Structural properties dictating selective optotracer detection of Staphylococcus aureus. Chembiochem. 2022;23. doi: 10.1002/cbic.202100684 PMC9400997 35298076

[64] Charych DH, Nagy JO, Spevak W, et al. Direct colorimetric detection of a receptor-ligand interaction by a polymerized bilayer assembly. Science. 1993;261(5121):585–588. doi: 10.1126/science.8342021 8342021

[65] Nilsson KPR, Inganäs O. Chip and solution detection of DNA hybridization using a luminescent zwitterionic polythiophene derivative. Nat Mater. 2003;2:419–424. doi: 10.1038/nmat899 12754497

[66] Doré K, Dubus S, Ho H-A, et al. Fluorescent polymeric transducer for the rapid, simple, and specific detection of nucleic acids at the zeptomole level. J Am Chem Soc. 2004;126(13):4240–4244. doi: 10.1021/ja038900d 15053613

[67] Klingstedt T, Nilsson KPR. Conjugated polymers for enhanced bioimaging. Biochim Biophys Acta. 2011;1810:286–296. doi: 10.1016/j.bbagen.2010.05.003 20471455

[68] Yang C, Huang H, Singh NM, et al. Synthetic conjugated Oligoelectrolytes are effective siRNA transfection carriers: relevance to pancreatic cancer gene therapy. Biomacromolecules. 2022;23(3):1259–1268. doi: 10.1021/acs.biomac.1c01498 35138828

[69] Nilsson KPR, Rydberg J, Baltzer L, et al. Self-assembly of synthetic peptides control conformation and optical properties of a zwitterionic polythiophene derivative. Proc Natl Acad Sci USA. 2003;100:10170–10174. doi: 10.1073/pnas.1834422100 12928490 PMC193534

[70] Nilsson KPR, Rydberg J, Baltzer L, et al. Twisting macromolecular chains: self-assembly of a chiral supermolecule from nonchiral polythiophene polyanions and random-coil synthetic peptides. Proc Natl Acad Sci USA. 2004;101:11197–11202. doi: 10.1073/pnas.0401853101 15280547 PMC509183

[71] Nilsson KPR, Herland A, Hammarström P, et al. Conjugated polyelectrolytes: conformation-sensitive optical probes for detection of amyloid fibril formation. Biochemistry. 2005; 44:3718–3724. doi: 10.1021/bi047402u 15751948

[72] Nilsson KPR, Hammarström P, Ahlgren F, et al. Conjugated polyelectrolytes–conformation-sensitive optical probes for staining and characterization of amyloid deposits. Chembiochem. 2006; 7:1096–1104. doi: 10.1002/cbic.200500550 16729336

[73] Sigurdson CJ, Nilsson KPR, Hornemann S, et al. Prion strain discrimination using luminescent conjugated polymers. Nat Methods. 2007;4:1023–1030. doi: 10.1038/nmeth1131 18026110

[74] Philipson O, Hammarström P, Nilsson KPR, et al. A highly insoluble state of Aβ similar to that of Alzheimer’s disease brain is found in Arctic APP transgenic mice. Neurobiol Aging. 2009;30:1393–1405. doi: 10.1016/j.neurobiolaging.2007.11.022 18192084

[75] Nilsson KPR, Ikenberg K, Aslund A, et al. Structural typing of systemic amyloidoses by luminescent-conjugated polymer spectroscopy. Am J Pathol. 2010;176:563–574. doi: 10.2353/ajpath.2010.080797 20035056 PMC2808065

[76] Herland A, Nilsson KPR, Olsson JDM, et al. Synthesis of a regioregular zwitterionic conjugated oligoelectrolyte, usable as an optical probe for detection of amyloid fibril formation at acidic pH. J Am Chem Soc. 2005;127:2317–2323. doi: 10.1021/ja045835e 15713111

[77] Nilsson KPR, Aslund A, Berg I, et al. Imaging distinct conformational states of amyloid-beta fibrils in Alzheimer’s disease using novel luminescent probes. ACS Chem Biol. 2007;2:553–560. doi: 10.1021/cb700116u 17672509

[78] Aslund A, Sigurdson CJ, Klingstedt T, et al. Novel pentameric thiophene derivatives for in vitro and in vivo optical imaging of a plethora of protein aggregates in cerebral amyloidoses. ACS Chem Biol. 2009;4:673–684. doi: 10.1021/cb900112v 19624097 PMC2886514

[79] Mahajan V, Klingstedt T, Simon R, et al. Cross β-Sheet conformation of Keratin 8 is a specific feature of Mallory–Denk bodies compared with other hepatocyte inclusions. Gastroenterology. 2011;141:1080–90.e7. doi: 10.1053/j.gastro.2011.05.039 21699779

[80] Klingstedt T, Aslund A, Simon RA, et al. Synthesis of a library of oligothiophenes and their utilization as fluorescent ligands for spectral assignment of protein aggregates†. Org Biomol Chem. 2011;9:8356–8370. doi: 10.1039/c1ob05637a 22051883 PMC3326384

[81] Klingstedt T, Shirani H, Åslund KOA, et al. The structural basis for optimal performance of oligothiophene-based fluorescent amyloid ligands: conformational flexibility is essential for spectral assignment of a diversity of protein aggregates. Chemistry. 2013;19:10179–10192. 23780508 10.1002/chem.201301463 PMC3884759

[82] Johansson LBG, Simon R, Bergström G, et al. An azide functionalized oligothiophene ligand–a versatile tool for multimodal detection of disease associated protein aggregates. Biosens Bioelectron. 2015;63:204–211. doi: 10.1016/j.bios.2014.07.042 25089818

[83] Simon RA, Shirani H, Aslund KOA, et al. Pentameric thiophene-based ligands that spectrally discriminate amyloid-β and tau aggregates display distinct solvatochromism and viscosity-induced spectral shifts. Chemistry. 2014;20:12537–12543. doi: 10.1002/chem.201402890 25111601 PMC4221846

[84] Klingstedt T, Shirani H, Mahler J, et al. Distinct spacing between anionic groups: an essential chemical determinant for achieving thiophene-based ligands to distinguish β-amyloid or tau polymorphic aggregates. Chemistry. 2015;21:9072–9082. doi: 10.1002/chem.201500556 26013403 PMC4517144

[85] Herrmann US, Schütz AK, Shirani H, et al. Structure-based drug design identifies polythiophenes as antiprion compounds. Sci Transl Med. 2015;7:299ra123. doi: 10.1126/scitranslmed.aab1923 26246168

[86] Shirani H, Linares M, Sigurdson CJ, et al. A palette of fluorescent thiophene-based ligands for the identification of protein aggregates. Chemistry. 2015;21:15133–15137. doi: 10.1002/chem.201502999 26388448 PMC4641461

[87] Lantz L, Shirani H, Klingstedt T, et al. Synthesis and characterization of thiophene-based donor-acceptor-donor heptameric ligands for spectral assignment of polymorphic amyloid-β deposits. Chemistry. 2020;26:7425–7432. doi: 10.1002/chem.201905612 32022335 PMC7318160

[88] Klingstedt T, Nilsson KPR. Luminescent conjugated poly- and oligo-thiophenes: optical ligands for spectral assignment of a plethora of protein aggregates. Biochem. 2012;40:704–710. doi: 10.1042/BST20120009 22817720

[89] Choong FX, Bäck M, Steiner SE, et al. Nondestructive, real-time determination and visualization of cellulose, hemicellulose and lignin by luminescent oligothiophenes. Sci Rep. 2016;6:35578. doi: 10.1038/srep35578 27759105 PMC5069672

[90] Choong FX, Bäck M, Fahlén S, et al. Real-time optotracing of curli and cellulose in live Salmonella biofilms using luminescent oligothiophenes. NPJ Biofilm Microb. 2016;2:16024. doi: 10.1038/npjbiofilms.2016.24 PMC5515270 28721253

[91] Butina K, Tomac A, Choong FX, et al. Optotracing for selective fluorescence-based detection, visualization and quantification of live S. aureus in real-time. NPJ Biofilm Microb. 2020;6:35. doi: 10.1038/s41522-020-00150-y PMC7547713 33037198

[92] Klingstedt T, Aslund A, Simon RA, et al. Synthesis of a library of oligothiophenes and their utilization as fluorescent ligands for spectral assignment of protein aggregates. Org Biomol Chem. 2011;9:8356–8370. 22051883 10.1039/c1ob05637a PMC3326384

[93] Weintraub A. Immunology of bacterial polysaccharide antigens. Carbohydr Res. 2003;338(23):2539–2547. doi: 10.1016/j.carres.2003.07.008 14670715

[94] Perchiacca JM, Ladiwala ARA, Bhattacharya M, et al. Structure-based design of conformation- and sequence-specific antibodies against amyloid β. Proc Natl Acad Sci USA. 2012;109:84–89. doi: 10.1073/pnas.1111232108 22171009 PMC3252897

[95] Groman RP. Chapter 93 - gram-positive infections. In: Silverstein DC Hopper K, editors. Small animal critical care medicine. 2nd ed. St. Louis: W.B. Saunders; 2015. p. 488–492. doi: 10.1016/B978-1-4557-0306-7.00093-3

[96] Scott RD. 2009. The direct medical costs of healthcare-associated infections in U.S. hospitals and the benefits of prevention.

[97] Idrees M, Sawant S, Karodia N, et al. Staphylococcus aureus biofilm: morphology, genetics, pathogenesis and treatment strategies. Int J Environ Res Public Health. 2021;18:7602. doi: 10.3390/ijerph18147602 34300053 PMC8304105

[98] Rohde M, Fischetti VA, Novick RP, et al. The gram-positive bacterial cell wall. Microbiol Spectr. 2019;7(3). doi: 10.1128/microbiolspec.GPP3-0044-2018 PMC11086966 31124431

[99] Vollmer W, Blanot D, de Pedro MA. Peptidoglycan structure and architecture. FEMS Microbiol Rev. 2008;32(2):149–167. doi: 10.1111/j.1574-6976.2007.00094.x 18194336

[100] Choong FX, Lantz L, Shirani H, et al. Stereochemical identification of glucans by a donor–acceptor–donor conjugated pentamer enables multi-carbohydrate anatomical mapping in plant tissues. Cellulose. 2019;26:4253–4264.

[101] Fey PD, Endres JL, Yajjala VK, et al. A genetic resource for rapid and comprehensive phenotype screening of nonessential Staphylococcus aureus genes. MBio. 2013;4(1):e00537–12. doi: 10.1128/mBio.00537-12 23404398 PMC3573662

[102] Asensio JL, Ardá A, Cañada FJ, et al. Carbohydrate-aromatic interactions. Acc Chem Res. 2013;46:946–954. doi: 10.1021/ar300024d 22704792

[103] van der Wal A, Norde W, Zehnder AJB, et al. Determination of the total charge in the cell walls of gram-positive bacteria. Colloids Surf. 1997;9:81–100. doi: 10.1016/S0927-7765(96)01340-9

[104] Costerton JW, Lewandowski Z, Caldwell DE, et al. Microbial biofilms. Annu Rev Microbiol. 1995;49(1):711–745. doi: 10.1146/annurev.mi.49.100195.003431 8561477

[105] Fux CA, Costerton JW, Stewart PS, et al. Survival strategies of infectious biofilms. Trends Microbiol. 2005;13(1):34–40. doi: 10.1016/j.tim.2004.11.010 15639630

[106] McCarty SM, Cochrane CA, Clegg PD, et al. The role of endogenous and exogenous enzymes in chronic wounds: a focus on the implications of aberrant levels of both host and bacterial proteases in wound healing. Wound Repair Regener. 2012;20(2):125–136. doi: 10.1111/j.1524-475X.2012.00763.x 22380687

[107] Serra DO, Hengge R. Stress responses go three dimensional - the spatial order of physiological differentiation in bacterial macrocolony. Biofilms Environ Microbiol. 2014;16:1455–1471. doi: 10.1111/1462-2920.12483 24725389 PMC4238805

[108] Donlan RM. Biofilms: microbial life on surfaces. Emerg Infect Dis. 2002;8:881–890. doi: 10.3201/eid0809.020063 12194761 PMC2732559

[109] Flemming H-C, Wingender J. The biofilm matrix. Nat Rev Microbiol. 2010;8(9):623–633. doi: 10.1038/nrmicro2415 20676145

[110] Flemming HC, Wingender J. Relevance of microbial extracellular polymeric substances (EPSs)–part I: structural and ecological aspects. Water Sci Technol. 2001;43:1–8. doi: 10.2166/wst.2001.0326 11381954

[111] Thongsomboon W, Serra DO, Possling A, et al. Phosphoethanolamine cellulose: a naturally produced chemically modified cellulose. Science. 2018;359(6373):334–338. doi: 10.1126/science.aao4096 29348238

[112] Römling U, Bian Z, Hammar M, et al. Curli fibers are highly conserved between Salmonella typhimurium and Escherichia coli with respect to operon structure and regulation. J Bacteriol. 1998;180(3):722–731. doi: 10.1128/JB.180.3.722-731.1998 9457880 PMC106944

[113] Antypas H, Choong FX, Libberton B, et al. Rapid diagnostic assay for detection of cellulose in urine as biomarker for biofilm-related urinary tract infections. NPJ Biofilm Microb. 2018;4(1):26. doi: 10.1038/s41522-018-0069-y PMC6203724 30393563

[114] Choong FX, Huzell S, Rosenberg M, et al. A semi high-throughput method for real-time monitoring of curli producing Salmonella biofilms on air-solid interfaces. Biofilm. 2021;3:100060. doi: 10.1016/j.bioflm.2021.100060 34841245 PMC8605384

[115] Pontes MH, Lee E-J, Choi J, et al. Salmonella promotes virulence by repressing cellulose production. Proc Natl Acad Sci USA. 2015;112(16):5183–5188. doi: 10.1073/pnas.1500989112 25848006 PMC4413311

[116] Corona-Izquierdo FP, Membrillo-Hernández J. A mutation in rpoS enhances biofilm formation in Escherichia coli during exponential phase of growth. FEMS Microbiol Lett. 2002;211:105–110. doi: 10.1111/j.1574-6968.2002.tb11210.x 12052558

[117] Gerstel U, Römling U. The csgD promoter, a control unit for biofilm formation in Salmonella typhimurium. Res Microbiol. 2003;154:659–667. doi: 10.1016/j.resmic.2003.08.005 14643403

[118] Bongomin F, Gago S, Oladele RO, et al. Global and multi-national prevalence of fungal diseases-estimate precision. J Fungi (Basel). 2017;3. doi: 10.3390/jof3040057 29371573 PMC5753159

[119] Mukaremera L, Lee KK, Mora-Montes HM, et al. Yeast, pseudohyphal, and hyphal morphogenesis differentially affects immune recognition. Front Immunol. 2017;8:629. doi: 10.3389/fimmu.2017.00629 28638380 PMC5461353

[120] Noble SM, Gianetti BA, Witchley JN. Candida albicans cell-type switching and functional plasticity in the mammalian host. Nature Rev Microbiol. 2017;15(2):96–108. doi: 10.1038/nrmicro.2016.157 27867199 PMC5957277

[121] Pierce CG, Vila T, Romo JA, et al. The Candida albicans biofilm matrix: composition, structure and function. J Fungi (Basel). 2017;3:14. doi: 10.3390/jof3010014 28516088 PMC5431293

[122] Kärkkäinen E, Jakobsson SG, Edlund U. Optotracing for live selective fluorescence-based detection of Candida albicans biofilms. Front Cell Infect Microbiol. 2022; 12:981454. doi: 10.3389/fcimb.2022.981454 36118028 PMC9478205

[123] Garcia-Rubio R, de Oliveira HC, Rivera J, et al. The fungal cell wall: Candida, cryptococcus, and aspergillus species. Front Microbiol. 2019;10:2993. doi: 10.3389/fmicb.2019.02993 31993032 PMC6962315

[124] Lowman DW, Ferguson DA, Williams DL. Structural characterization of (1→3)-β-d-glucans isolated from blastospore and hyphal forms of Candida albicans. Carbohydr Res. 2003;338(14):1491–1496. doi: 10.1016/S0008-6215(03)00169-1 12829394

[125] Flemming H-C, Wuertz S. Bacteria and archaea on earth and their abundance in biofilms. Nat Rev Microbiol. 2019;17(4):247–260. doi: 10.1038/s41579-019-0158-9 30760902

[126] Dragoš A, Kovács ÁT. The peculiar functions of the bacterial extracellular matrix. Trends Microbiol. 2017;25:257–266. doi: 10.1016/j.tim.2016.12.010 28089324

[127] Nadell CD, Drescher K, Wingreen NS, et al. Extracellular matrix structure governs invasion resistance in bacterial biofilms. ISME J. 2015;9(8):1700–1709. doi: 10.1038/ismej.2014.246 25603396 PMC4511925

[128] Yin W, Wang Y, Liu L, et al. Biofilms: the microbial “protective clothing” in extreme environments. Int J Mol Sci. 2019;20(14):3423. doi: 10.3390/ijms20143423 31336824 PMC6679078

[129] Høiby N, Bjarnsholt T, Givskov M, et al. Antibiotic resistance of bacterial biofilms. Int J Antimicrob Agents. 2010;35:322–332. doi: 10.1016/j.ijantimicag.2009.12.011 20149602

[130] Ito A, Taniuchi A, May T, et al. Increased antibiotic resistance of Escherichia coli in mature biofilms. Appl Environ Microbiol. 2009;75(12):4093–4100. doi: 10.1128/AEM.02949-08 19376922 PMC2698376

[131] Stewart PS, Costerton JW. Antibiotic resistance of bacteria in biofilms. Lancet. 2001;358(9276):135–138. doi: 10.1016/S0140-6736(01)05321-1 11463434

[132] Eckert JA, Rosenberg M, Rhen M, et al. An optotracer-based antibiotic susceptibility test specifically targeting the biofilm lifestyle of Salmonella. Biofilms. 2022;4:100083. doi: 10.1016/j.bioflm.2022.100083 PMC9474290 36117547

[133] Cimdins A, Simm R. Semiquantitative analysis of the red, dry, and rough colony morphology of Salmonella enterica serovar typhimurium and Escherichia coli using Congo red. Methods Mol Biol. 2017;1657:225–241. 28889298 10.1007/978-1-4939-7240-1_18

[134] Kostakioti M, Hadjifrangiskou M, Hultgren SJ. Bacterial biofilms: development, dispersal, and therapeutic strategies in the dawn of the postantibiotic era. Cold Spring Harb Perspect Med. 2013;3(4):a010306. doi: 10.1101/cshperspect.a010306 23545571 PMC3683961

[135] Chakraborty P, Bajeli S, Kaushal D, et al. Biofilm formation in the lung contributes to virulence and drug tolerance of mycobacterium tuberculosis. Nat Commun. 2021;12(1):1606. doi: 10.1038/s41467-021-21748-6 33707445 PMC7952908

[136] Ross P, Mayer R, Benziman M. Cellulose biosynthesis and function in bacteria. Microbiol Rev. 1991;55(1):35–58. doi: 10.1128/mr.55.1.35-58.1991 2030672 PMC372800

[137] Saxena IM, Brown RM. Biosynthesis of cellulose. In: Morohoshi N, Komamine A, editors. Progress in biotechnology. Vol. 18, Elsevier; 2001. p. 69–76. doi: 10.1016/S0921-0423(01)80057-5

[138] Terlizzi ME, Gribaudo G, Maffei ME. UroPathogenic Escherichia coli (UPEC) infections: virulence factors, bladder responses, antibiotic, and non-antibiotic antimicrobial strategies. Front Microbiol. 2017;8:1566. doi: 10.3389/fmicb.2017.01566 28861072 PMC5559502

[139] Costerton JW, Stewart PS, Greenberg EP. Bacterial biofilms: a common cause of persistent infections. Science. 1999;284(5418):1318–1322. doi: 10.1126/science.284.5418.1318 10334980

[140] Choong FX, Bäck M, Schulz A, et al. Stereochemical identification of glucans by oligothiophenes enables cellulose anatomical mapping in plant tissues. Sci Rep. 2018;8:3108. doi: 10.1038/s41598-018-21466-y 29449697 PMC5814555

[141] Lakowicz JR. Quenching of fluorescence. In: Principles of fluorescence spectroscopy. Boston, MA: Springer US; 2006. p. 277–330.

[142] Boerjan W, Ralph J, Baucher M. Lignin biosynthesis. Annu Rev Plant Biol. 2003;54:519–546. doi: 10.1146/annurev.arplant.54.031902.134938 14503002

[143] Uses of lignin. [accessed 2023 July 3]. https://www.valmet.com/pulp/other-valueadding-processes/lignin-extraction/lignin-uses/

[144] Ahmad N, Zakaria MR. Oligosaccharide From Hemicellulose. In: Sapuan SM, Hassan MA, editors. Lignocellulose for future bioeconomy. Elsevier; 2019. p. 135–152. doi: 10.1016/B978-0-12-816354-2.00008-6

[145] Huang L-Z, Ma M-G, Ji X-X, et al. Recent developments and applications of hemicellulose from wheat straw: a review. Front Bioeng Biotechnol. 2021;9:690773. doi: 10.3389/fbioe.2021.690773 34239863 PMC8258147

[146] Wahlström N, Nylander F, Malmhäll-Bah E, et al. Composition and structure of cell wall ulvans recovered from Ulva spp. along the Swedish west coast. Carbohydr Polym. 2020;233:115852. doi: 10.1016/j.carbpol.2020.115852 32059903

[147] Tan ECD, Lamers P. Circular bioeconomy concepts—a perspective. Front Sustain. 2021;2. doi: 10.3389/frsus.2021.701509

